# A New Quasi‐Solid Polymer Electrolyte for Next‐Generation Na–O_2_ Batteries: Unveiling the Potential of a Polyamide‐Polyether System

**DOI:** 10.1002/advs.202504490

**Published:** 2025-05-23

**Authors:** Mohamed Yahia, Marina Enterría, Cristina Pozo‐Gonzalo, Nagore Ortiz‐Vitoriano

**Affiliations:** ^1^ Center for Cooperative Research on Alternative Energies (CIC energiGUNE) Basque Research and Technology Alliance (BRTA) Alava Technology Park Albert Einstein 48 Miñano 01510 Spain; ^2^ Institute for Frontier Materials Deakin University Geelong Victoria 3200 Australia; ^3^ Instituto de Carboquímica (ICB‐CSIC) C/Miguel Luesma Castán, 4 Zaragoza 50018 Spain; ^4^ Ikerbasque Basque Foundation for Science María Díaz de Haro 3 Bilbao 48013 Spain

**Keywords:** galvanostatic measurements, ionic conductivity, Na–O_2_ battery, Pebax1657, polyethylene oxide, quasi‐solid polymer electrolyte (QSPE), quasi‐solid‐state battery (QSSB)

## Abstract

A novel quasi‐solid polymer electrolyte (QSPE) composed of polyamide (PA) and polyethylene oxide (PEO), commercially known as Pebax1657, and combined with 1 M sodium bis(trifluoromethanesulfonyl)imide (NaTFSI) in diethylene glycol dimethyl ether (diglyme, DEGDME), has been investigated for sodium–oxygen (Na–O_2_) batteries. Pebax1657 QSPE exhibits high ionic conductivity (6.57 × 10^−4^ S cm^−1^ at room temprerature ‐ RT), an oxidation onset potential of 4.69 V versus Na/Na⁺, and an enhanced Na⁺ transference number (t_Na_^⁺^ ≈ 0.40). Structural analysis (Raman spectroscopy, differential scanning calorimetry, X‐ray diffraction, small‐angle X‐ray scattering) confirms reduced PEO crystallinity and formation of orderly nanodomains, facilitating Na⁺ transport. Long‐term galvanostatic cycling in Na|Na symmetrical cells demonstrates stable overpotentials (≈80 mV) at 75 µA cm⁻^2^ for 210 h, outperforming conventional liquid electrolytes (≈110 h). Pebax1657 QSPE enables higher discharge capacities (2.60 mAh cm⁻^2^ at 75 µA cm⁻^2^; 2.11 mAh cm⁻^2^ at 150 µA cm⁻^2^) with lower overpotentials (≈0.2 V). It sustains 25 cycles at 75 µA cm⁻^2^ and 35 cycles at 150 µA cm⁻^2^ at 0.25 mAh cm⁻^2^, with a Coulombic Efficiency (CE) of 80–90%. Compared to the state of the art, Pebax1657 QSPE offers improved electrochemical stability, lower overpotentials, and better capacity retention. Its sustainability and versatility make it a strong candidate for Na–O_2_ batteries and other energy storage applications.

## Graphical abstract

This study introduces a novel, sustainable Pebax1657‐based quasi‐solid polymer electrolyte (QSPE) that enhances the performance and safety of sodium–oxygen (Na–O_2_) batteries. The developed QSPE shows improved Na^+^ ion transport, reduced overpotentials, and better cycling stability compared to the conventional liquid systems, highlighting its potential for next‐generation energy storage applications, including high‐performance Na‐ion and Na–O_2_ batteries.

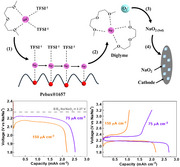

## 1 Introduction

The development of sustainable energy storage systems is a critical challenge in the transition to a low‐carbon economy. Among various energy storage technologies, metal‐air/O_2_ batteries have emerged as a promising alternative to lithium‐ion batteries (LIBs) due to their high theoretical energy density, environmental friendliness, and cost‐effectiveness.^[^ ^1^ ^]^ Among the different chemistries, Na–O_2_ batteries are particularly attractive, offering an energy density nearly six times higher than that of LIBs.^[^ ^1a–c^ ^]^ Additionally, sodium (Na) is the sixth most abundant element in the Earth's crust, providing a significant advantage in terms of resource availability and cost. Since the development of room‐temperature Na–O_2_ batteries by Sun^[^ ^2^ ^]^ and Hartmann,^[^ ^3^ ^]^ the research community has primarily focused on understanding the key working and failure mechanisms during cycling, guiding the development of new electrode and electrolyte materials.^[^ ^1^, ^4^ ^]^ Among these, the electrolyte plays a significant role in determining the overall electrochemical performance, efficiency, and lifespan of Na–O_2_ batteries. An ideal electrolyte must possess suitable ionic conductivity, high thermal stability, and resistance to chemical degradation against reactive oxygen species generated during battery operation.

Scientists are still actively exploring stable electrolytes capable of sustaining long‐term battery operation; however, significant advancements have been made in this field. Ether‐based electrolytes and ether/ionic liquid (IL) hybrid electrolytes have emerged as the preferred choices for non‐aqueous Na–O_2_ batteries.^[^ ^1^, ^5^ ^]^ However, liquid electrolytes pose significant safety concerns, including leakage, volatility, and flammability, which hinder their practical application. These challenges could be addressed through the implementation of solid‐state electrolytes. Beyond improving safety, solid‐state electrolytes also help mitigate issues commonly associated with metal‐based energy storage technologies, such as dendrite growth and crossover contaminants (e.g., H_2_O) which can lead to metallic Na passivation and battery failure during cycling.^[^ ^1^ ^]^ Additionally, for practical deployment, solid‐state electrolytes should exhibit ease of processability, cost‐effectiveness, and environmental sustainability.^[^ ^6^ ^]^ However, despite their potential, the application of solid‐state electrolytes in Na–O_2_ batteries remains largely underexplored, mainly due to the relative novelty and mechanistic complexity of these systems compared to metal‐ion technologies.^[^ ^1a–c^ ^]^

Quasi‐solid‐state electrolytes (QSSEs), including polymer‐based and gel electrolytes (QSPEs), as well as solid‐state electrolytes (SSEs) such as ceramic and polymer nanocomposite electrolytes, have been extensively investigated for energy storage applications.^[^ ^1^, ^7^ ^]^ QSPEs composed of a polymer matrix embedded with a liquid electrolyte‐stand out due to their enhanced safety, mechanical stability, and ionic conductivity compared to traditional liquid electrolytes.^[^ ^1^ ^]^ Several studies have demonstrated the advantages of QSPEs in sodium‐based systems. In 2019, Ha et al.,^[^ ^8^ ^]^ introduced an ionogel composed of an ionic liquid and polymer matrix as a protective layer for the Na metal anode. This ionogel effectively prevented side reactions and improved both CE and overpotential compared to using an ionic liquid alone (CE: 100 vs 25%). Similarly, Wang et al.^[^ ^9^ ^]^ developed a QSPE composed of Polyvinylidene fluoride (PVDF)/NaClO_4_/SiO_2_/Tetraethylene glycol dimethyl ether, which exhibited high ionic conductivity (1.0 mS cm^−1^), non‐flammability, and moisture resistance, while also promoting uniform Na deposition and NaO_2_ formation.^[^ ^9^ ^]^ In another study, He et al.^[^ ^10^ ^]^ reported on a QSPE composed of PVDF‐hexafluoropropylene (HFP)@Nafion that exhibited good flame resistance, liquid retention, and hydrophobicity, leading to excellent cycling stability at relatively high current densities (80 cycles at 1000 mA g^−1^ and 1000 mAh g^−1^). However, despite these benefits, PVDF‐based polymer electrolytes present significant drawbacks due to their high fluoride content and susceptibility to degradation when exposed to reactive superoxide anions.^[^ ^11^ ^]^ The cleavage of C─F bonds results in the degradation of the polymer, compromising mechanical stability and generating harmful byproducts.^[^ ^12^ ^]^ Furthermore, Iputera et al.^[^ ^13^ ^]^ successfully developed a QSPE by utilizing poly(ethylene oxide) as the polymer matrix, incorporating an electrolyte based on NaTFSI and nanosized sodium superionic conductor powder (NZSP) to modulate the electrochemical impedance and ion conduction of the polymer thin film.

While PEO‐based electrolytes have been widely explored in Na‐based batteries,^[^ ^14^ ^]^ they still suffer from low Na⁺ transference numbers (typically < 0.25) and poor ionic conductivity due to the high crystallinity of PEO and the low concentration of free Na⁺. As a result, recent polymer electrolyte strategies aim to enhance Na⁺ transport by tuning interactions between metal ions and the polymer backbone.

A promising alternative is Pebax1657, an innovative thermoplastic elastomer composed of rigid PA blocks and flexible PEO segments in a 40:60 wt.% ratio. At room temperature, PA segments are glassy, providing mechanical strength, while PEO domains are rubbery, facilitating Na⁺ transport.^[^ ^15^ ^]^ This unique combination allows alkaline metals salts to dissolve, enabling effective Na^+^ conduction due to the PEO domains, while the hard PA domains provide physical crosslinking points via abundant hydrogen bonds, significantly improving electrolyte mechanical stability. Pebax1657 is widely used in gas separation technologies due to its dense morphology,^[^ ^15^, ^16^ ^]^ and it also presents a more sustainable alternative to fully synthetic polymers. Pebax polymers, particularly the bio‐based Pebax Rnew series, have attracted recent attention for their sustainability due to their polyamide 11 (PA11) content derived from renewable castor oil.^[^ ^16^ ^]^ Compared to conventional petroleum‐based polymers, Pebax Rnew reduces reliance on fossil fuels, and hence contributing to lower environmental footprint. Moreover, adopting green solvent systems like water/ethanol mixtures in membrane fabrication reduces commonly use hazardous solvents such as NMP, reinforcing Pebax's alignment with sustainable manufacturing practices. While life cycle assessment (LCA) studies specific to Pebax are limited due to its novelty, broader LCA analyses of bio‐based polymers suggest a significant reduction in carbon emissions and nonrenewable energy consumption compared to fossil‐based materials.^[^ ^17^ ^]^ This study highlights Pebax's potential in next‐generation energy storage systems. Although comprehensive LCA studies for Pebax in battery applications are still needed, existing research strongly supports its role in advancing sustainable energy solutions through bio‐derived content, electrochemical stability, and recyclability.

In summary, the chemical and morphological uniqueness of Pebax1657 should control oxygen permeability, facilitate Na^+^ transport while enhancing the mechanical strength of the final electrolyte. Herein, we develop a novel sustainable QSPE based on the Pebax1657 membrane embedded with 1 M NaTFSI in diglyme for Na–O_2_ batteries. This QSPE demonstrates significant advantages over conventional liquid electrolytes using Celgard separators, offering enhanced Na metal protection, improved electrochemical stability, and superior Na⁺ transport.

In this manuscript, the proposed QSPE has been tested as a proof of concept in both symmetric Na cells and full Na–O_2_ cells, delivering stable cycling performance. These findings highlight the potential of advanced polymer electrolytes to drive energy storage innovation, offering a safer, more efficient, and sustainable solution for next‐generation Na–O_2_ batteries.

## 2 Results and Discussion

### 2.1 Physicochemical Characterization of Polymer Membranes

First, the Pebax1657 membranes were synthesized after dissolving Pebax1657 pellets in an EtOH/H_2_O mixture as mentioned in the experimental section, then the membranes were first characterized and compared to Celgard H2010 separators, without any liquid electrolyte. The morphology and physicochemical properties of both polymers were analyzed using scanning electron microscopy (SEM), Brunauer‐Emmett‐Teller (BET) adsorption isotherms, electrolyte uptake, Gurley air permeability, and conductivity measurements, providing a comprehensive comparison of their structural and functional differences.

The SEM micrographs reveal distinct morphological differences between the two materials. The Celgard separator exhibits a porous structure with interconnected large pores of average size ≈0.5 ± 0.2 µm (Figure S2a, Supporting Information). In contrast, the Pebax1675 membrane presents a dense, fibrous structure with no visible large pores (Figure S2b, Supporting Information), making it particularly suitable for applications requiring controlled oxygen permeability.^[^ ^16^, ^19^ ^]^ Therefore, we envision that these differences in porosity and structure could significantly impact Na–O_2_ battery performance. A low‐porosity membrane is particularly advantageous as it helps suppress dendritic growth, reducing the risk of sodium penetration through the pores and thereby preventing short circuits, ultimately enhancing battery safety and longevity.

Air permeability is a crucial parameter for evaluating membrane porosity and was assessed using the Gurley air permeability test, following the standardized methodology described in the experimental section (Figure S2c, Supporting Information). The Gurley number represents the time (in seconds) required for a fixed volume of air to pass through the membrane, where a lower value indicates higher air permeability, corresponding to a more porous structure. The results showed that Celgard exhibits a significantly lower Gurley number (234.5 s) compared to Pebax (966.2 s), confirming its greater air permeability and higher porosity in Celgard, which aligns with the SEM analysis (Figures S2a,b, Supporting Information) demonstrating the dense structure of Pebax1657.

Furthermore, the N_2_ physisorption analysis (Figure S2d, Supporting Information), based on BET theory, was performed to examine pore dimensions and specific surface area (SSA). The analysis revealed a significantly lower SSA for Pebax1657 membrane (6 m^2^ g^−1^) compared to Celgard separator (26 m^2^ g^−1^). The N_2_ adsorption isotherms further demonstrate that Pebax1657 does not exhibit any micro‐ or mesopores across the pressure range studied, confirming its dense non‐porous structure. On the other hand, Celgard shows adsorption characteristics consistent with wide mesopores, particularly at high relative pressures (P/P_o_ > 0.8), suggesting the presence of larger pores as shown in the SEM micrographs.

Beyond structural differences, the electrolyte uptake and ionic conductivity are also critical parameters, as they directly influence the mechanical stability of the electrolyte and its mass transport properties, ultimately impacting the electrochemical performance of the battery. The electrolyte uptake (1 M NaTFSI /diglyme), shown in Figure S3a (Supporting Information), reveals that the Pebax1657 membrane exhibits a slightly higher electrolyte uptake percentage (50.78 ± 1.20%) compared to Celgard (45.39 ± 1.51%). It should be noted that, considering the lower porosity of the Pebax1657 membrane, a comparable amount of electrolyte to Celgard was absorbed. Besides the intrinsic porosity and permeability of a given membrane, chemical interactions between the polymer and the electrolyte also affect electrolyte uptake. PEO and PA are reported to absorb electrolyte by chemical interaction due to their strong affinity for polar solvents and ionic species, even in relatively dense structure.^[^ ^20^ ^]^ Thus, the functional groups in Pebax could interact with the electrolyte components, enhancing the membrane's ability to absorb and retain the electrolyte.

The Nyquist plots in Figure S3b (Supporting Information) illustrates the ionic conductivity of the Pebax1657 membrane and Celgard separator, both incorporating the same volume of 1 M NaTFSI/diglyme. For clarity, these systems will hereafter be referred to as Celgard/Liquid electrolyte (L.E.) and Pebax1657 QSPE, respectively. The achieved ionic conductivity is (6.57 × 10^−4^ ± 1.46 × 10^−4^ S cm^−1^) for Pebax1657 QSPE compared to (7.24 × 10^−4^ ± 2.69 × 10^−4^ S cm^−1^) for Celgard/L.E., both are within the same order of magnitude, but the ionic conductivity of Pebax1657 QSPE is slightly lower than that of Celgard/L.E., which is consistent with the slightly higher impedance observed for Pebax1657 QSPE (24.19 ± 5.16 Ω) compared to Celgard/L.E. (15.09 ± 4.9 Ω), as shown in Figure S3 (Supporting Information). The ionic conductivity of Pebax 1657 membrane is comparable to that of modified PEO‐based solid polymer electrolytes, which typically range between 10^−4^ and 10^−3^ S cm^−1^, depending on factors such as temperature, salt concentration, and the addition of plasticizers or fillers.^[^ ^13^, ^15^, ^21^ ^]^ Additionally, the PVDF‐HFP‐based electrolytes used in Na–O_2_ batteries^[^ ^10^, ^22^ ^]^ achieved ionic conductivities in the range of 10^−3^ S cm^−1^, further demonstrating the suitability of our system for solid‐state Na–O_2_ batteries.^[^ ^10^, ^13^ ^]^ Figure S3c (Supporting Information) presents a comparative analysis of electrolyte uptake and ionic conductivity for both systems, highlighting the balance between electrolyte absorption and ion transport properties, which are key parameters in optimizing electrolyte performance for Na–O_2_ batteries.

### 2.2 Chemical Stability and Interaction of Pebax1657 Membrane with Electrolyte

The chemical stability of the Pebax1657 QSPE was assessed through nuclear magnetic resonance spectroscopy (NMR): ^1^H‐NMR, ^23^Na‐NMR, and ^19^F‐NMR. After 48 h of immersion, the membrane was removed, and the remaining liquid electrolyte was subjected to liquid NMR analysis. The ^1^H‐NMR spectra peak assignments are showed in Figure S4 and Table S1 (Supporting Information). In summary, the peaks do not show any signals related to Pebax1657 membrane, confirming that the electrolyte does not dissolve or degrade the polymer. Additionally, ^23^Na and ^19^F NMR spectra also show peaks corresponding exclusively to the electrolyte, further confirming the stability of the Pebax1657 membrane in the electrolyte environment, which is crucial for efficient and long‐term operation.

**Figure 1:**
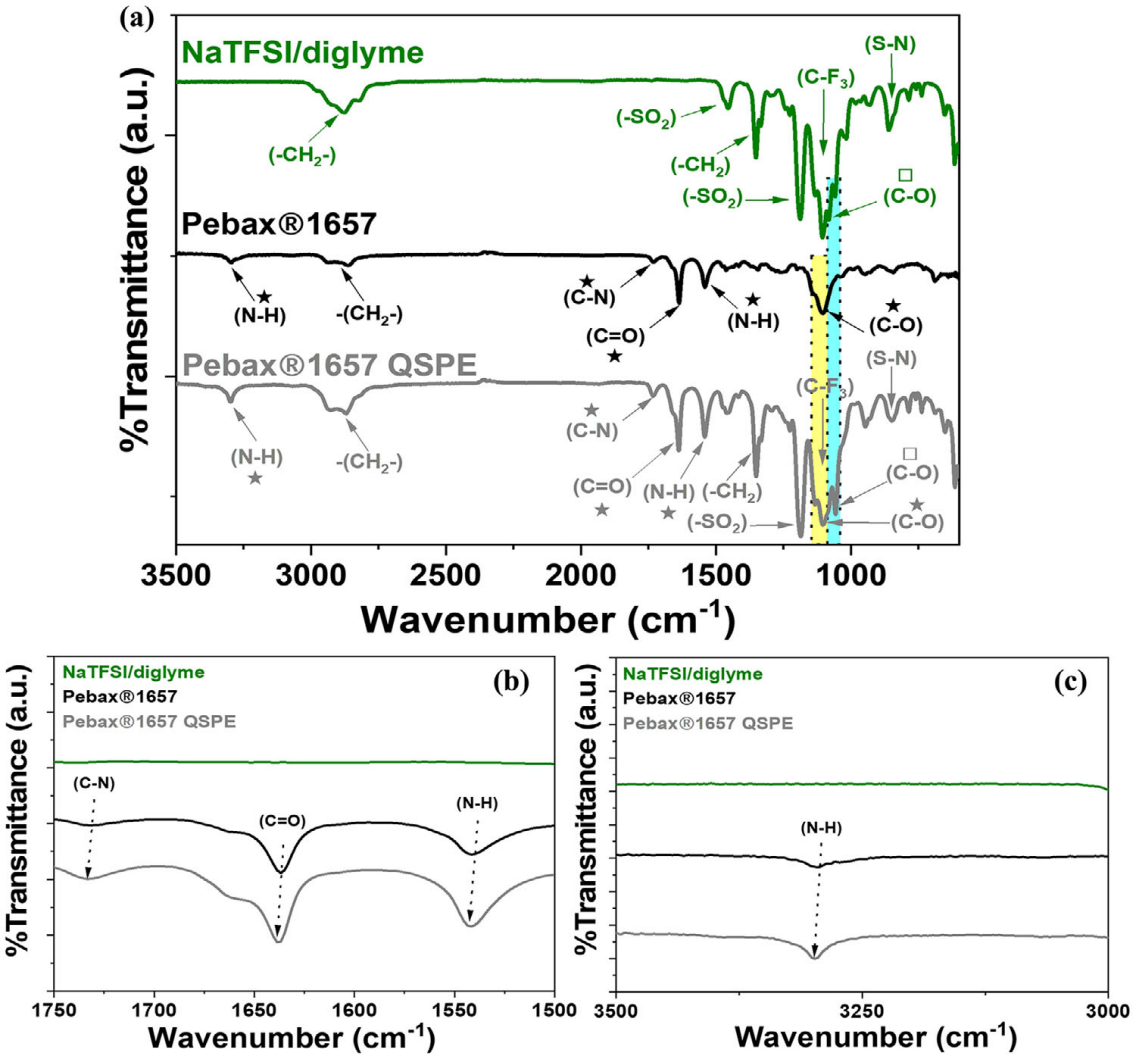
Attenuated total reflectance (ATR)‐FTIR spectra of Pebax1657 membrane, NaTFSI/diglyme, and Pebax1657 QSPE (Pebax1657 membrane was immersed in 1 M NaTFSI/diglyme and dried before analysis).

Fourier transform infrared spectroscopy (FTIR) (**Figure** **1**) of the Pebax1657 QSPE was performed to investigate possible interactions between the membrane and the electrolyte, providing further insights into the mechanisms influencing liquid electrolyte uptake (Figure 1c). As shown in Figure 1a‐b, the FTIR spectra of the Pebax1657 QSPE, along with the with Pebax1657 membrane and NaTFSI/diglyme, were analyzed to identify specific peaks associated with each material, allowing for a detailed examination of their interactions. Figure 1a and Table S2 (Supporting Information) show characteristic peaks within the Pebax1657 structure (Figure 1a) corresponding to N─H stretching, CH_2_ stretching, N─H bending, C═O stretching around, C─N stretching, and C─O stretching. These peaks confirm the existence of PEO and PA segments in line with the previous published studies.^[^ ^20^ ^]^ Furthermore, the FTIR spectrum of NaTFSI/diglyme displays distinct peaks associated with the electrolyte components. For the diglyme solvent, the characteristic peaks correspond to CH_2_ stretching, ─CH_2_ bending, and C─O stretching, and for NaTFSI salt, S═O (sulfonyl group) asymmetric and symmetric stretching, CF_3_ stretching, and S─N stretching, respectively, which are in line with previously published studies.^[^ ^15^, ^23^ ^]^ Upon incorporating NaTFSI/diglyme electrolyte into the Pebax1657 membrane, the FTIR spectrum of the resulting Pebax1657 QSPE shows shifts in key peaks associated with the Pebax1657 polymer matrix, indicating specific interactions between the polymer functional groups and the electrolyte components. Specifically, the peaks associated to the PA segments, the C═O peak shifts from 1635 to 1640 cm^−1^, the C─N peak shifts from 1725 to 1733 cm^−1^, and the N‐H peaks shift from 1535 to 1541 cm^−1^ (bending mode) and from 3294 to 3300 cm^−1^ (stretching mode). Changes in the C─O vibration band, characteristic of the PEO segments, are less clear due to overlapping with peaks from the electrolyte; however, a shift from 1098 to 1104 cm^−1^ is observed. It is well established that in solid PEO‐based electrolytes, metal cation transport primarily occurs through complexation and decomplexation of ether oxygen groups.^[^ ^23a^ ^]^

Thus, these peak shifts, considering the resolution of the equipment, suggest interactions between the functional groups in the Pebax1657 membrane matrix and the NaTFSI salt within the electrolyte.^[^ ^15^, ^23^ ^]^ This chemical interaction may explain the high electrolyte uptake, despite the dense structure of the Pebax1657 membrane.

Raman spectroscopy was employed to investigate the Na⁺ solvation and transport behavior in four different environments: liquid NaTFSI/diglyme electrolyte, Pebax1657 membrane, and Pebax1657 QSPE, and Pebax1657 QSPE after galvanostatic cycling (plating/stripping), as shown in Figure S5 and Table S3 (Supporting Information). In the liquid state, several sharp peaks can be assigned to TFSI⁻:≈740, 1240 and 1260 cm^−1^, corresponding to S─N─S, CF_3_, and SO_2_ vibrational bands, respectively.^[^ ^24^ ^]^ Additionally, the spectrum reveals the presence of Na⁺‐diglyme complexes with abundant free TFSI ^−^anions, along with characteristics vibrational bands of free diglyme, such as C‐O‐C stretch at ≈845 cm^−1^ and ‐CH_2_ bending mode at ≈1450 cm^−1^.^[^ ^25^ ^]^ Upon incorporation of the electrolyte into the Pebax1657 membrane, band broadening^[^ ^14^, ^26^ ^]^ and slight shifts to higher values were observed, indicating possible interaction of Na⁺ with polar functional groups (C─O, C─N, and C═O) within the Pebax polymer backbone. This coordination is further supported by slightly shifts to higher values in the characteristic peaks of the electrolyte components. The TFSI⁻ vibration bands at ≈740, 1240 and 1260 cm^−1^, corresponding to S─N─S, CF_3_, and SO_2_ vibrational bands, respectively,^[^ ^24^ ^]^ become broadened upon electrolyte incorporation. Similarly, the diglyme bands the C─O─C stretch at ≈845 cm^−1^ and ─CH_2_ bending at 1450 cm^−1^, also show noticeable broadening, suggesting interaction with Na⁺. In the case of Pebax1657 QSPE,^[^ ^27^ ^]^ characteristic C─O stretching bands are slightly shifted and broadened from ≈830, 1060, 1126, and 1280 cm^−1^ to 850, 1065, 1135, and 1287 cm^−1^, respectively. The C─N stretching band shifts from ≈1445 to 1450 cm^−1^, while the C─N bending mode shows significant broadening around ≈930 cm^−1^. Additionally, the C═O stretching band is also broadened, centered around ≈1640 cm^−1^. In the case of Pebax1657 QSPE after galvanostatic cycling (plating/stripping), the Raman analysis revealed a significant broadening or diminished of the Pebax 1657 polymer‐related vibrational bands associated with C─O, C─N, and C═O groups, suggesting structural rearrangement or a loss of vibrational order within the Pebax matrix. Additionally, the diglyme C─O─C vibrational band at ≈852 cm^−1^ is markedly diminished, and the TFSI⁻ band corresponding to the S─N─S vibration shifts to lower value and broadens from ≈743 to 737 cm^−1^, accompanied by reduced intensity or broadening across all TFSI⁻ vibrational bands (S‐N‐S, CF_3_, and SO_2_). These spectral changes underscore the active involvement of Pebax functional groups in facilitating Na^+^ transport, likely through transient coordination interactions that enable Na^+^ hopping. This behavior may contribute to the enhanced ionic mobility and improved electrochemical performance observed during cycling. Overall, the Raman analysis supports the existence of a multi‐step Na^+^ conduction mechanism and confirms the critical role of Pebax1657 QSPE in regulating solvation dynamics and interfacial processes in Na–O_2_ batteries.

Figure S6 (Supporting Information) illustrates the possible Na^+^ ions transport mechanism and interactions with the PA and PEO segments in the Pebax1657 QSPE.^[^ ^23^, ^28^ ^]^ Although, the crystalline regions of PEO typically hinder both ionic conductivity and transport of Na^+^,^[^ ^28^ ^]^ in the case of the Pebax1657 QSPE, the presence of PA segments could contribute to a reduction in PEO crystallinity. This effect has been observed in other polymer systems such as PEO‐polyurethane (PU),^[^ ^23a^ ^]^ where the presence of N‐H and O‐H groups in PA promotes strong hydrogen bonding with PEO segments. Initially, Na⁺ ions are fully solvated in the liquid electrolyte region by diglyme, forming Na⁺ diglyme complexes stabilized by the coordination of ether oxygen atoms,^[^ ^24^ ^]^ as shown in Figure S6 (Supporting Information). Upon entering the Pebax1657 membrane, partial desolvation of Na⁺ occurs as diglyme molecules are replaced or complemented by coordination with the polar functional groups of the Pebax polymer, namely the ether linkages (─C─O─C─) groups. These groups provide transient coordination sites, effectively forming a hopping mechanism through the polymer matrix, where Na⁺ ions migrate by sequential association and dissociation along the polar sites of the polymer backbone. After crossing the Pebax QSPE, Na⁺ ions undergo complete desolvation at the polymer/O_2_ cathode interface, a critical step before participating in the electrochemical reaction. Here, the desolvated Na⁺ ions react with superoxide (O_2_^−^) generated from dissolved oxygen, forming NaO_2_ as the primary discharge product. NaO_2_ initially forms as a solvated intermediate and then precipitates as a solid phase on the porous carbon cathode, ensuring stable NaO_2_ deposition without passivating the cathode surface too early.

The Celgard separator was also characterized by FTIR analysis (Figure S7a and Table S2, Supporting Information), showing the characteristic peaks of polypropylene.^[^ ^29^ ^]^ The FTIR spectrum showed the prominent peaks corresponding to the C─H stretching vibrations of methylene (CH_2_) groups, CH_3_ symmetric deformation, and CH_2_ bending modes, respectively.^[^ ^29^ ^]^ The FTIR spectrum of Celgard with NaTFSI/diglyme electrolyte showed the combined distinct peaks associated with both materials (Celgard and electrolyte, Figure S7, Supporting Information). No significant shifts were observed, indicating the absence of chemical interactions with the NaTFSI/diglyme electrolyte components. This suggests that the role of Celgard is primarily as an inert physical separator.

**Figure 2:**
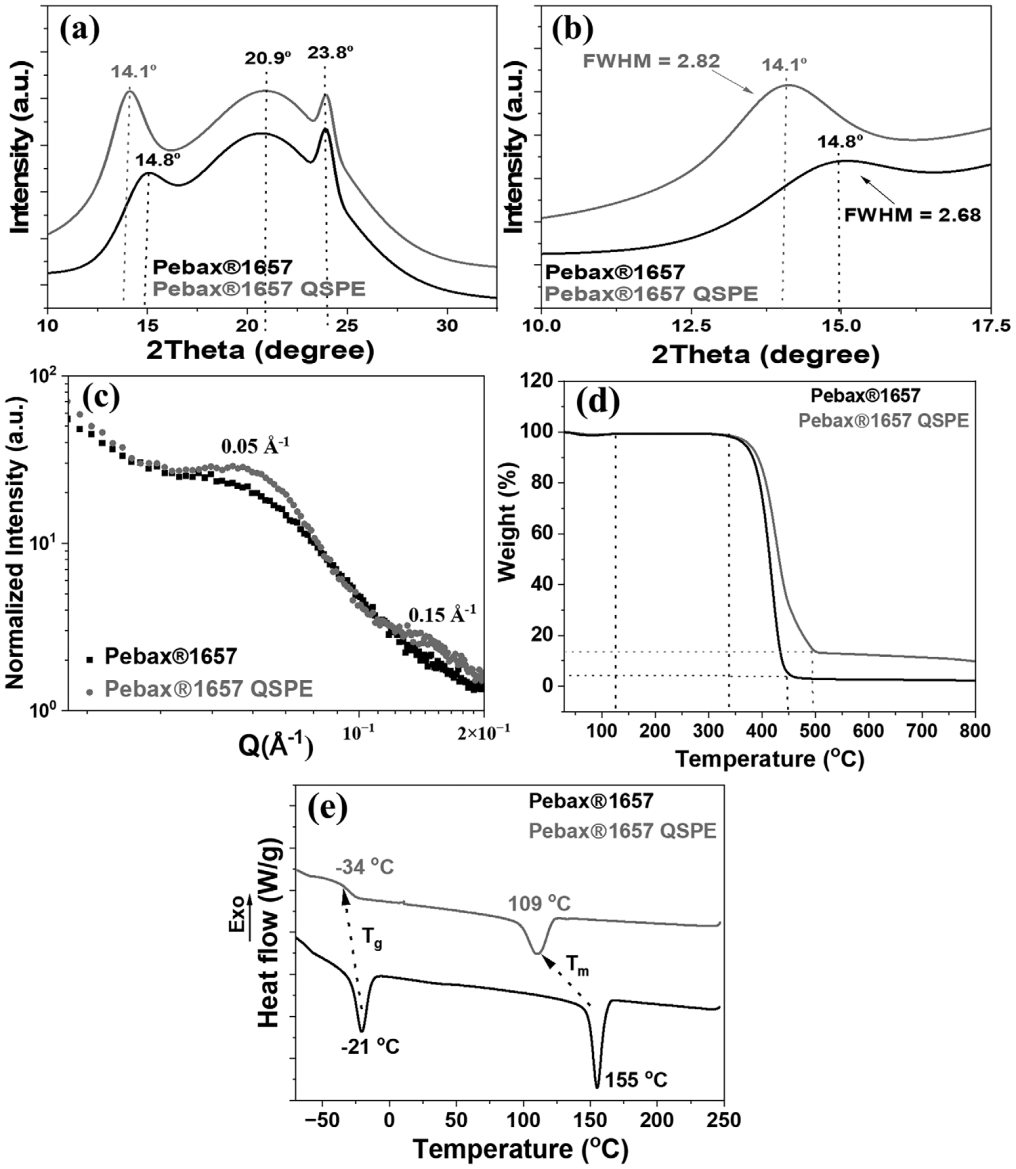
XRD patterns a) and the FWHM b), Small‐angle X‐ray Scattering (SAXS) analysis for the electrolytes c), thermogravimetric analysis (TGA) up to 800 °C, 10 °C min^−1^ d), and Differential Scanning Calorimetry (DSC) analysis with a heating rate of 10 °C min^−1^ over a temperature range from −80 to 250 °C for Pebax1657 membrane and Pebax1657 QSPE impregnated with NaTFSI/diglyme e).

**Figure** **2**a represents the X‐ray diffraction (XRD) patterns of the Pebax1657 membrane and the Pebax1657 QSPE to provide further insight into possible structural changes induced by the integration of the electrolyte into the membrane (Figure 2a). The XRD pattern reveals distinct diffraction peaks, indicating the presence of crystalline regions within the Pebax1657 membrane, in agreement with previous reports.^[^ ^15^, ^23^ ^]^ Specifically: i) at 2θ = 14.8° (d = 4.15 Å) corresponds to crystalline regions formed by PA blocks, ii) at 2θ = 20.9° (d = 3.70 Å) reflects the regular arrangement of polymeric chains, possibly from PEO segments or mixed regions, and iii) at 2θ = 23.8° (d = 3.34 Å) corresponds to densely packed crystalline domains within the PEO segments.^[^ ^15^, ^27^ ^]^ Upon introducing the NaTFSI/diglyme mixture into the Pebax1657, the XRD pattern shows a notable shift of the diffraction peak at 2θ = 14.8° to 14.1°, along with an increase in relative peak intensity. This can be attributed to an expansion of the lattice structure and decrease in the crystallinity of the polymer.^[^ ^15^, ^23^ ^]^ Interestingly, only the diffraction peak corresponding to the crystalline regions formed by PA blocks seems to be affected by the presence of the electrolyte. The Full Width at Half Maximum (FWHM) corresponding to Pebax1657 membrane is 2.68, while for the Pebax1657 QSPE, the FWHM increases to 2.82^[^ ^15^, ^23^ ^]^ (Figure 2b). A sharper and more well‐defined peak suggests a higher degree of crystallinity and larger grain size. However, it is important to note that the FWHM can be influenced by various factors, including instrumental broadening, sample preparation, and crystal defects. Therefore, refined XRD peak fitting were performed by applying the Pseudo‐Voigt function, which is a linear combination of the Gaussian and Lorentzian functions for accurate determination of the FWHM (Figure S8, Supporting Information).

Furthermore, the XRD analysis of the Celgard separator was performed as a control study. Figure S7b (Supporting Information) reveals typical crystalline peaks for polypropylene^[^ ^30^ ^]^ at 2θ = 14.1° (d = 6.27 Å), 16.8° (d = 5.27 Å), 18.5° (d = 4.79 Å), 21.3° (d = 4.17 Å), and 23.9° (d = 3.72 Å), corresponding to the crystalline α‐phase of isotactic polypropylene.^[^ ^30^ ^]^ After the incorporation of NaTFSI/diglyme, no shift or changes were detected in the positions or intensities of the XRD peaks, further confirming that the crystalline structure of Celgard remains unaffected by the incorporation of the NaTFSI/diglyme electrolyte.

To further analyze the nanoscale internal structure and organization of Pebax structure, SAXS was conducted on both the Pebax1657 membrane and the Pebax1657 QSPE (Figure 2c). The Pebax1657 membrane exhibits a prominent scattering peak ≈0.05 Å^−1^, indicating the presence of PEO soft segments,^[^ ^15^, ^31^ ^]^ which self‐organize to form nanodomains through the Pebax1657 membrane.^[^ ^15^, ^31^, ^32^ ^]^ As shown in Figure 2c, when the electrolyte (NaTFSI/diglyme) is incorporated into the membrane, the SAXS profile changes noticeably and the normalized intensity of the Pebax1657 QSPE increases gradually. Furthermore, the scattering peak (0.05 Å^−1^) for PEO becomes slightly higher in intensity and a new scattering peak at 0.15 Å^−1^ emerges, suggesting the formation of more orderly nanodomains compared to the pristine Pebax1657 membrane. These enhanced nanodomains create favorable pathways, which can facilitate Na^+^ transport within the dense membrane,^[^ ^15^, ^31^, ^33^ ^]^ and better ionic conductivity in comparison with neat PEO.^[^ ^15^, ^31^, ^33^ ^]^

To measure the thermal stability and composition of the samples, TGA of the Pebax1657 membrane and the Pebax1657 QSPE was conducted (Figure 2d). Both materials exhibit three primary degradation stages: 80–120, 120–330, and 330–450 °C for Pebax1657 membrane and broader (330–500 °C) for the Pebax1657 QSPE. Figure 5d shows that the Pebax1657 membrane undergoes a total weight loss of ≈95%, associated with the decomposition of PA blocks,^[^ ^15^, ^27^ ^]^ while the Pebax1657 QSPE exhibits a slightly lower total weight loss of ≈85%. This reduced weight loss at higher temperatures suggests that the incorporation of the electrolyte enhances the overall thermal stability of the Pebax1657 membrane.

Figure 2e represents the DSC of the Pebax1657 membrane and the Pebax1657 QSPE. The thermograms indicate that the Pebax1657 membrane exhibits two distinct thermal transitions: a glass transition temperature (T_g_) at −21 °C followed by a melting temperature (T_m_) at 155 °C, corresponding to the crystalline PA phase.^[^ ^15^, ^16^, ^34^ ^]^ A second T_m_ corresponding to PEO chains should also been observed as reported in the literature, however the transformation of crystalline PEO into amorphous phase in Pebax1657 would explain the absence of that T_m_. Upon the incorporation of NaTFSI salt and diglyme, T_g_ decreases to a lower value (−34 °C) which suggests a plasticizing effect of the electrolyte, and enhanced mobility of the polymer chains.^[^ ^15^, ^21^ ^]^ Additionally, the melting temperature (T_m_) of the Pebax1657 QSPE system also shifts to a lower temperature (109 °C) which suggests a disruption of the regular packing of the polymer chains.^[^ ^15^, ^21^ ^]^ The reduced crystallinity of the polymer matrix implies a higher degree of amorphous regions which can further promote ion transport.^[^ ^15c^ ^]^ This is consistent with the findings of the XRD.

A cross‐section of the Pebax1657 QSPE was studied to assess the homogenous integration of the electrolyte within the membrane (Figure S9a, Supporting Information). The EDS‐SEM mapping (Figure S9b,c, Supporting Information) confirms the uniform distribution of key electrolyte elements (Na, F, S) within the QSPE, which is crucial for Na–O_2_ battery performance.

### 2.3 Ionic Transport Properties and Electrochemical Stability of the Pebax1657 QSPE

Linear Sweep Voltammetry (LSV) was performed to evaluate the electrochemical stability of Celgard/L.E. and Pebax1657 QSPE (Figure S10a, Supporting Information). The onset of the anodic process was determined as the potential at which a significant current density of 15 µA cm^−2^ was observed. In the case of Celgard/L.E. that value corresponds to 4.28 V versus Na/Na⁺ and in contrast, the Pebax1657 QSPE shows a slightly higher oxidation onset potential of ≈4.69 V versus Na/Na⁺ (an increase of 0.39 V) (Figure S10a, Supporting Information). As previously reported, the oxidation cut‐off potential of a typical Na–O_2_ battery can reach up to 4.5 V, so conventional liquid electrolytes, like diglyme and tetraglyme, would decompose at that potential. The enhanced oxidation stability in the case of Pebax1657 QSPE is likely due to the presence of less free diglyme solvent due to the chemical interactions with the polymer, as opposed to the Celgard separator where the electrolyte is only adsorbed on the surface.

The Na^+^ transference number (t_Na₊_), a crucial transport parameter for fast charge‐discharge performance, was measured for the Celgard/L.E. and The Pebax1657 QSPE using the Bruce–Vincent method and applying Equation 3. Detailed calculations can be found in the Supporting Information. Figure S10b,c (Supporting Information) exhibits the initial and steady‐state impedance responses and the chronoamperometry (CA) for both the Celgard/L.E. and the Pebax1657 QSPE using symmetric Na/Na cells.

For Celgard/L.E., the t_Na₊_ is 0.32 whereas Pebax1657 QSPE exhibits a higher transference number (t_Na₊_ ∼ 0.40). This value surpasses the typical range observed in traditional PEO‐based electrolytes (t_Na₊_ < 0.25),^[^ ^14^, ^35^ ^]^ indicating enhanced Na⁺ transport. The increase in t_Na₊_ for Pebax1657 QSPE is attributed to the reduction in polymer crystallinity and the formation of orderly nanodomains upon electrolyte incorporation, which facilitates more efficient Na⁺ migration within the polymer matrix.

**Figure 3:**
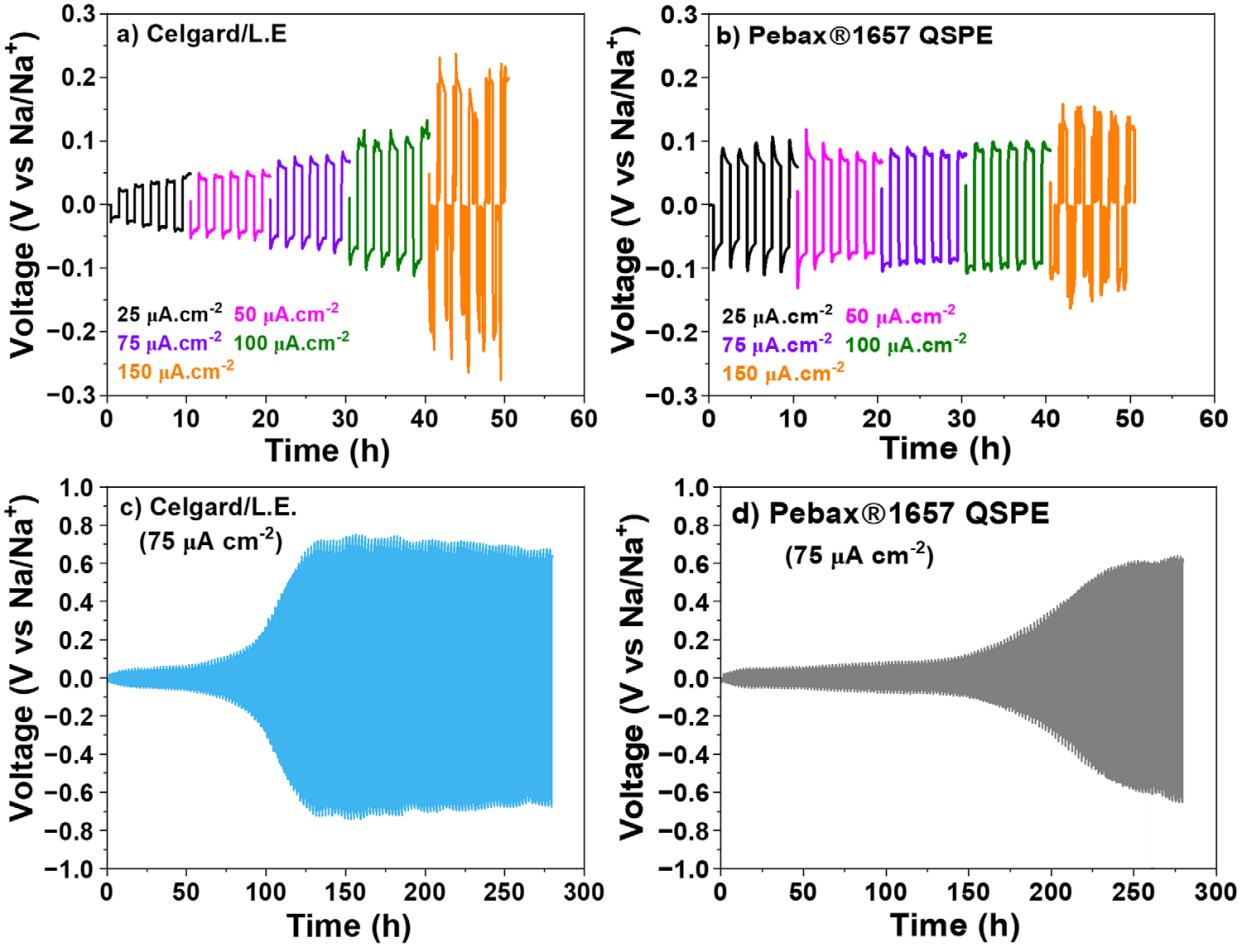
Voltage profiles of Na|Na symmetrical cells using Celgard/L.E. a) and Pebax1657 QSPE b), along with long‐term galvanostatic cycling of Na symmetrical cells at a current density of 75 µA cm^−2^ for Celgard/L.E. c) and Pebax1657 QSPE d). The cells underwent OCV measurements for 30 min, followed by cycling at room temperature for 5 cycles with a 2‐h step time at various current densities (25–150 µA cm^−2^).

Plating/stripping experiments were conducted to evaluate the electrochemical performance of sodium symmetrical cells. **Figure** **3** compares the galvanostatic cycling behavior of cells using Celgard/L.E. (a) and Pebax1657 QSPE (b), at current densities ranging from 25 to 150 µA cm^−2^. As shown in Figure 3a, the cells using Celgard demonstrate stable cycling at low current densities (25 and 50 µA cm^−2^) with relatively low overpotentials (≈50 mV). As the current density increases to 75 and 100 µA cm^−2^, a noticeable rise in overpotential is observed, approximately doubling to ≈100 mV. At 150 µA cm^−2^, the cells experience higher overpotential increase to ≈250 mV followed by battery failure. At high current densities, localized hotspots may form, allowing sodium to penetrate the pores, which could lead to a potential short circuit in the cell.^[^ ^21^, ^36^ ^]^

In contrast, Pebax1657 QSPE (Figure 3b) exhibits more consistent performance across the tested current densities. At 25 and 50 µA cm^−2^, the cells show stable cycling with overpotentials ≈80 mV, and at higher current densities (75 and 100 µA cm^−2^) the overpotential increases slightly to ≈100 mV, similar to the Celgard‐based cells. In addition, at 150 µA cm^−2^, the cells also fail after a few cycles, similar to Celgard, but the overpotential is slightly lower than Celgard (≈150 mV vs 250 mV). Furthermore, Figure 3c,d presents the long‐term galvanostatic cycling of Na symmetrical cells at 75 µA cm^−2^ using Celgard and Pebax1657 QSPE, respectively. Celgard shows a significant increase in overpotential over time, suggesting that its porous structure may lead to irregular Na⁺ ion distribution.^[^ ^9^ ^]^ Specifically, after ≈110 h, the overpotential rises sharply from ≈50 to 700 mV. In contrast, Pebax1657 QSPE maintains a stable overpotential of ≈80 mV for more than 210 h, demonstrating superior electrochemical stability. The lower overpotential observed in the Pebax1657 QSPE probably indicates a more stable interfacial layer, reducing resistance and suppressing the formation of localized hotspots during cycling.

Figure S11 (Supporting Information) illustrates the short‐term and long‐term cycling stages for both Celgard/L.E. (Figure S11a,c, Supporting Information) and Pebax1657 QSPE (Figure S11b,d, Supporting Information). During the short‐term stage (10–20 h), both systems exhibit stable cycling with a low overpotential of ≈30 mV. However, in the long‐term stage (270–280 h), Celgard shows a notable increase in overpotential, reaching ≈700 mV, whereas Pebax1657 QSPE maintains a slightly lower overpotential (≈600 mV), highlighting its superior interfacial stability and more uniform Na^+^ ion transport.

### 2.4 Electrochemical Testing of Full Na–O_2_ Batteries

**Figure 4:**
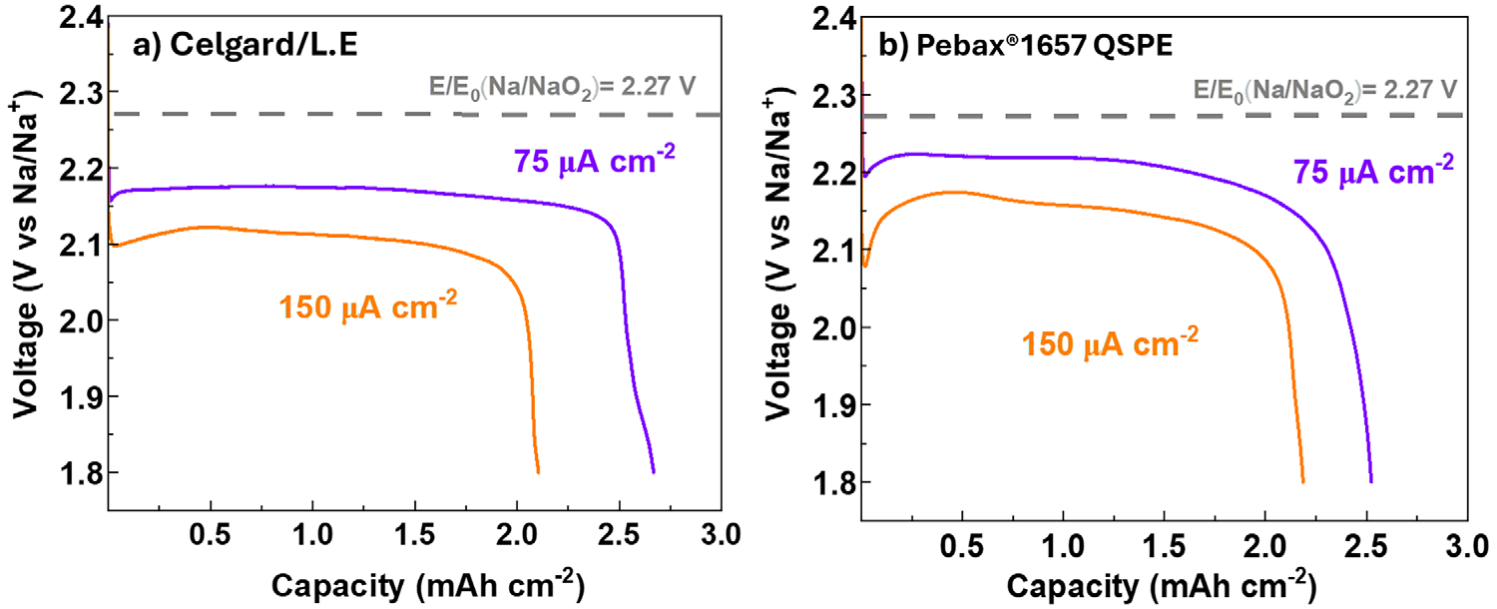
Galvanostatic deep‐discharge curves at different current densities for the Swagelok‐type Na–O_2_ batteries assembled using a) Celgard/L.E. and b) Pebax1657 QSPE. The measurements were performed at RT.

**Figure** **4** presents the galvanostatic deep‐discharge curves for Swagelok‐type Na–O_2_ batteries assembled with Celgard/L.E. and Pebax1657 QSPE. Both systems yield comparable discharge capacities, decreasing slightly by increasing the current density from 75 to 150 µA cm^−2^. In the Celgard/L.E. system, the capacity decreases from 2.53 to 2.19 mAh cm^−2^ (Figure 4a), whereas in the Pebax1657 QSPE, it decreases from 2.60 to 2.11 mAh cm^−2^ (Figure 4b).

Considering the thermodynamic potential for NaO_2_ formation (2.27 V vs Na/Na^+^, indicated by the dotted grey line on Figure 4), the discharge overpotential in the Celgard‐based battery remains stable within the range of 100–160 mV, depending on the applied current density. In contrast, the Pebax1657 QSPE system maintains a discharge plateau significantly closer to the theoretical potential, with an overpotential of only 50–110 mV overpotential. This is a key factor in battery performance, as a lower overpotential translates to minimal energy loss and improved specific power density.^[^ ^37^ ^]^

The higher discharge voltage (i.e., enhanced kinetics) for NaO_2_ growth in the Pebax1657 QSPE battery could be attributed to a complex and favorable interplay among free sodium ion concentration, solvent availability and oxygen solubility.

To determine the chemistry of the discharge products, Raman spectroscopy was performed (Figure S12, Supporting Information). The prominent peak at 1156 cm^−1^, observed in all the spectra, is characteristic of NaO_2_ formation, strongly suggesting that NaO_2_ is the primary discharge product,^[^ ^8^, ^38^ ^]^ regardless of the applied current or electrolyte. Furthermore, the Raman spectra exhibit two distinct peaks at ≈1350 and 1580 cm^−1^, corresponding to the D and G bands of the carbon cathode electrode, respectively. These peaks are associated with the disordered (D band) and the graphitic (G band) carbon structures within the electrode composition.^[^ ^39^ ^]^ The Raman spectra do not show significant peaks ≈800–1085 or 1340 cm^−1^ associated with the presence of carbonate (CO_3_^2−^) and formate (HCOO^−^) by‐products, respectively. The absence of these peaks further supports the high selectivity toward NaO_2_ formation and the lack of common by‐products, which are typically found in ether‐based electrolytes.^[^ ^8^, ^38^, ^39^ ^]^ The Raman analysis confirms the absence of impurities and demonstrates the formation of NaO_2_ as the main discharge product in both electrolyte systems and different current densities. Consequently, the difference in cell polarization as a function of the electrolyte arises from variations in nucleation and/or reaction mechanisms.

**Figure 5:**
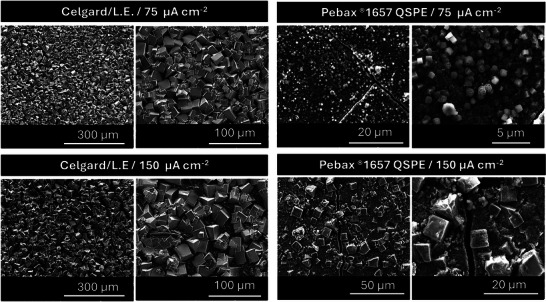
SEM imaging of the cathode/oxygen interface after discharge at different current densities; a,b) 75 µA cm^−2^ and c,d) 150 µA cm^−2^, for the Swagelok‐type Na–O_2_ batteries assembled using a–c) Celgard/L.E. b–d) Pebax1657 QSPE.

The discharge product morphology at the oxygen/cathode interface after deep discharge was studied by SEM imaging (**Figure** **5**). The NaO_2_ discharge products in the Celgard‐based cells (Figure 5a–c) exhibit large cubic morphologies at both current densities. At 75 µA cm^−2^, the NaO_2_ cubes display a broad size distribution of small and large cubes ranging from ≈10 to 20 µm, with a high population of the smaller cubes (≈10 µm). When the current density increases to 150 µA cm^−2^, the main particle size exhibits a slight increase, particularly for the larger particles, which expand within the range of 10 to 25 µm. In contrast, the Pebax1657 QSPE discharge cells exhibit a decrease in NaO_2_ particle size. At 75 µA cm^−2^, the NaO_2_ particles are significantly smaller (ranging from 1 to 3 µm, Figure 5b) and are more uniformly distributed. As the current increases to 150 µA cm^−2^, the particle size grows to a range of 5–10 µm, while maintaining a uniform distribution. The distinct morphology and distribution of the discharge products, together with the significantly lower surface coverage of the cathode observed by SEM imaging, suggests a more controlled and growth process when using the Pebax1657 QSPE, compared to the conventional liquid electrolyte with a Celgard separator.

In summary, this study has showed that the discharge product analysis by SEM reveals that Pebax1657 QSPE produces significantly smaller and more uniformly distributed NaO_2_ particles (1–3 µm at 75 µA cm^−2^, 5–10 µm at 150 µA cm^−2^), compared to Celgard/L.E. (10‐20 µm and 10–25 µm, respectively). This controlled particle growth reduces cathode passivation, enhancing discharge capacity and cycle life.

The solubility of O_2_ and/or the NaO_2_ species formed through oxygen reduction reaction (ORR) dictates the discharge mechanism and the battery kinetics. In contrast, the Pebax1657 QSPE discharge cells exhibit a decrease in NaO_2_ particle size. At 75 µA cm^−2^, the NaO_2_ particles are much smaller (ranging from 1 to 3 µm, Figure 5b) and more uniformly distributed. As the current density increases to 150 µA cm^−2^, the particle size grows to a range of 5–10 µm, while maintaining a uniform distribution. It has been demonstrated that the size of NaO_2_ cubes is directly proportional to the solubility of NaO_2_ in the electrolyte system and inversely proportional to the sodium concentration in the electrolyte.^[^ ^24^ ^]^ This behavior can be attributed to the fact that, in metal‐air batteries, the discharge process is predominantly governed by a solution‐mediated mechanism, where the reduced species dissolved into the electrolyte, enabling nucleation and growth within the solution phase before precipitating onto the cathode surface. The morphology and size variation of the discharge products arises from the nucleation/growth mechanism, which is influenced by the interaction between discharge intermediates and the solvent. Controlling the discharge product morphology and size requires a careful balance between the solvent's ability to solvate and desolvate NaO_2_ intermediates. Beyond solvent effects, the sodium salt concentration in liquid electrolytes has also been reported to significantly impact NaO_2_ particle size.^[^ ^24^, ^40^ ^]^ The solubility of NaO_2_ follows a volcano‐shaped trend, reaching its maximum at intermediate Na^+^ concentrations (1–1.5 M) due to the interplay of two competing effects; 1) increased Na^+^ activity stabilizing superoxide ions, and 2) reduced free solvent availability, limiting NaO_2_ solvation. As a result, the size of NaO_2_ cubes in this work mirrors this solubility trend, with the largest particles forming in the Celgard/L.E. system, where electrolyte composition and porosity promote greater NaO_2_ solubility and larger discharge products.

Based on published studies, the confinement of the liquid electrolyte within the polymer matrix, and/or its interaction with the polymer structure limits the solubility of O_2_ and NaO_2_ intermediates, primarily due to low availability of free solvent molecules, as observed in the QSPE system. This reduced solubility impacts the nucleation and growth process of discharge products, differentiating QSPE‐based systems from conventional liquid electrolytes. Furthermore, Na⁺ ion diffusion in the QSPE system likely follows multiple transport pathways, influenced by its hybrid structure, which integrates both solid‐like and liquid‐like phases. In the solid‐like regions, Na^+^ ions interact with the functional groups in the polymer matrix, hopping between adjacent coordination sites.^[^ ^41^ ^]^ In contrast, within the liquid‐phase domains, Na⁺ ions are solvated by the free solvent molecules, enabling diffusion through these liquid pockets via a typical liquid‐state mechanism.^[^ ^42^ ^]^ Additionally, SAXS experiments reveal the formation of orderly nanodomains upon integration of the liquid electrolyte within the Pebax1657 membrane. These nanodomains create favorable pathways that facilitate Na^+^ transport through the dense membrane, resulting in a better ionic conductivity in comparison with neat Pebax polymer.

The greater solubility of O_2_ and NaO_2_ in the Celgard/L.E. system facilitates the growth of larger NaO_2_ cubes (Figure 5a–c). In contrast, in the Pebax1657 QSPE cell, the limited solvent availability restricts NaO_2_ solubility, leading to smaller, more uniformly distributed discharge particles (Figure 5b–d) due to slower nucleation and growth rates. The uniform distribution of the discharge products in the QSPE system could also be attributed to a more homogeneous flux of Na^+^ and the slightly higher transference number (t_Na⁺_ ≈ 0.40 in Pebax 1657 QSPE versus t_Na⁺_ ≈ 0.32 in Celgard/L.E., as shown in Figure S10, Supporting Information). However, at higher current densities, the inhomogeneity of the discharge product and the slight increase in NaO_2_ particle size may be linked to an increased demand for Na^+^ transport.^[^ ^43^ ^]^ Beyond a certain current density threshold, the liquid‐phase domains in the QSPE may become the primary transport medium, compensating for the limitations in ion mobility within the solid‐like regions.

The cathode/electrolyte interface, corresponding to the carbon fibers, was examined by SEM imaging (Figure S13a, Supporting Information). In the Celgard‐based cells, NaO_2_ deposits were observed on the carbon fibers, though in lower amounts compared to the cathode/oxygen interface. In contrast, in the Pebax1657 QSPE system, no NaO_2_ deposits were detected at the cathode‐electrolyte interface at either current density (Figure S13b,d, Supporting Information).

As the oxygen interface becomes passivated by deposition of discharge products, it gradually becomes electronically insulating and unreactive toward the ORR. Consequently, the reaction shifts to regions where electronic conduction is still possible, allowing ORR to continue and maintain the reaction rate typically near the electrolyte side of the electrode. The Pebax1657 QSPE system facilitates the formation of well‐distributed NaO_2_ particles, only a few microns in size, which helps prevent passivation of the oxygen interface. As a result, NaO_2_ formation remains confined to the cathode surface rather than dispersing into the electrolyte bulk, with no particles accumulating along the gas diffusion layer. This behavior contributes to a more stable reaction environment, ensuring a high‐voltage discharge plateau and enhanced electrochemical performance.

**Figure 6:**
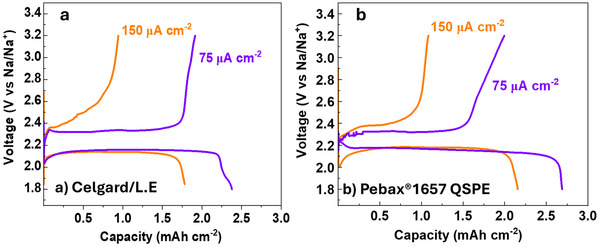
Galvanostatic discharge/charge curves (Full cyclability) at current densities (75 and 150 µA cm^−2^) for the Swagelok‐type Na–O_2_ batteries assembled using a) Celgard/L.E. and b) Pebax1657 QSPE. CEis denoted for each profile. The measurements were performed at room temperature.

**Figure** **6** presents the full discharge/charge curves for batteries assembled with Celgard/L.E. and Pebax1657 QSPE at 75 and 150 µA cm^−2^. The charge curves reveal significant differences in overpotential between the two systems. At low current densities, the charge overpotential is similar for both electrolytes (160–180 mV). However, at high current densities, the Celgard‐based battery exhibits a much larger charge overpotential compared to the QSPE‐based system (460 vs 250 mV). Increasing the current density from 75 to 150 µA cm^−2^ resulted in a significant drop of CE in both electrolyte systems from ≈80 to ≈50%. This could be ascribed to the restricted availability of solvent molecules to redissolve back the oxidized NaO_2_ solid products during charge. Additionally, the larger size and increased inhomogeneity of the discharge products generated at higher current density may further contribute to this effect (Figure 5c‐d). At high currents, this phenomenon becomes more pronounced, as the solvation rate may not be sufficient to keep up with the rapid oxidation process, leading to incomplete redissolution of the discharge products and ultimately lower CE.

**Figure 7:**
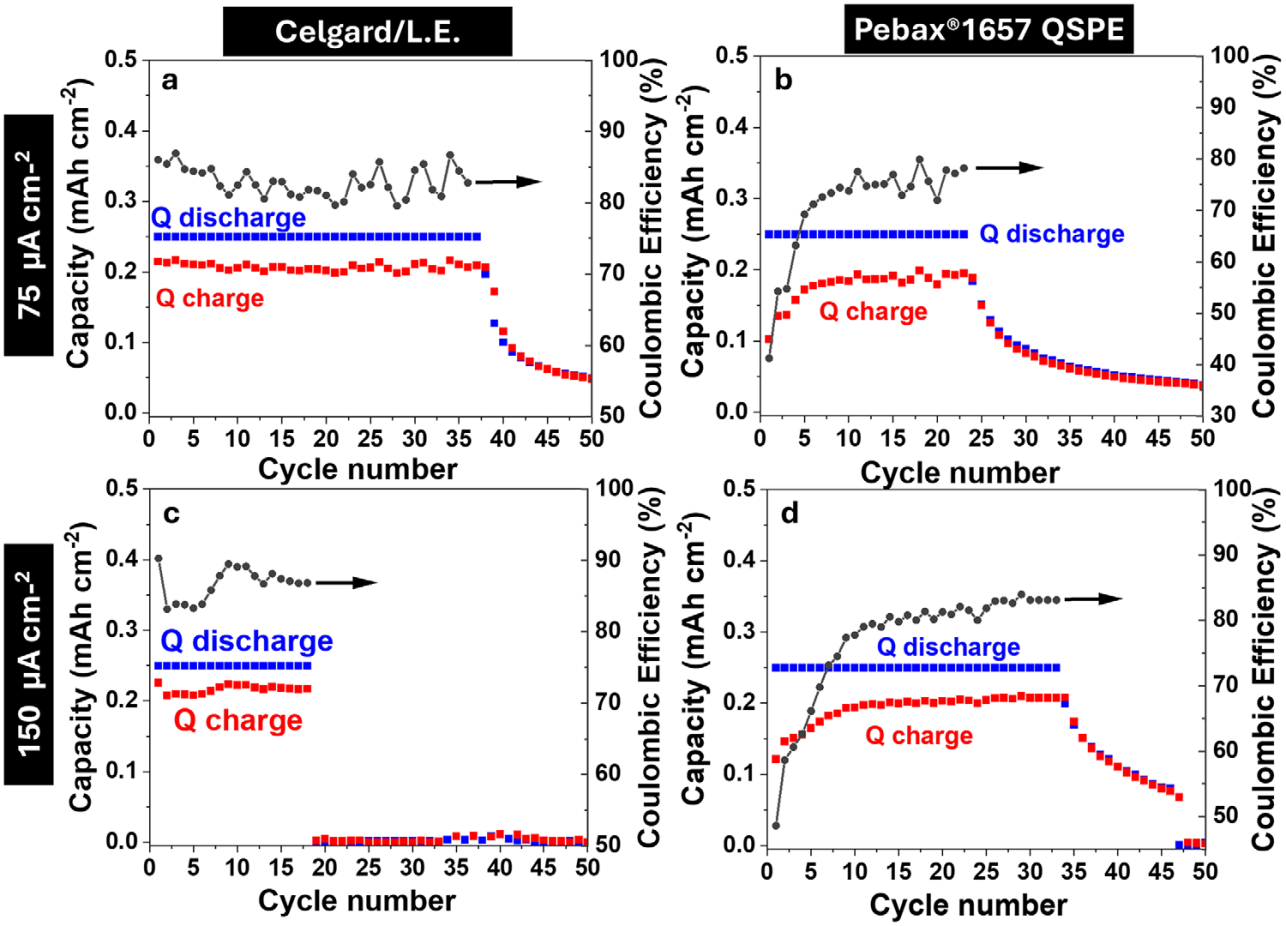
Evolution of discharge capacity (left axis, points denoted as red squares), charge capacity (left axis, points denoted as blue squares), and CE (right axis, points denoted as grey circles) as a function of the number of cycles, with a limited capacity of 0.25 mAh cm^−2^ and a cut‐off potential of 1.8 V (a–d). All measurements were performed at RT.

In order to assess the cyclability of both systems, shallow cycling tests (i.e., limited capacity set to 0.25 mAh cm^−2^) were conducted for Celgard/L.E. and Pebax1657 QSPE, under two different current densities (75 and 150 µA cm^−2^) (**Figure** **7**; Figure S14, Supporting Information). At 75 µA cm^−2^, the Celgard/L.E. full cell (Figure 7a) delivers 40 cycles before experiencing progressive capacity fading, with a CE ranging between 80 and 90%, indicating initial stability but substantial degradation after extended cycling. In contrast, the full cell using Pebax1657 QSPE delivered around 25 cycles (Figure 7b). Interestingly, a lower CE is observed in the initial cycles, but it recovers from around cycle 10 onward, stabilizing between 75 and 85%.

At a higher current density of 150 µA cm^−2^, the Celgard‐based battery (Figure 7c) shows a significantly shortened cycle life, delivering only 20 cycles before experiencing a sharp decline in performance but the C.E. remains between 80 and 90%. On the other hand, the Pebax1657 QSPE (Figure 7d) exhibits notably extended cycling performance, sustaining 35 cycles at high current density. During the first 10 cycles, the CE remains lower (≈78%), but it gradually increases to 83% for the remaining cycles, following a similar trend to its behavior at lower current density.

Furthermore, Figure S14e (Supporting Information) shows the charge overpotential evolution for Celgard/L.E. and Pebax1657 QSPE at 75 and 150 µA cm^−2^, respectively. Celgard/L.E. maintains a stable overpotential of ≈150 mV at 75 µA cm^−2^ and 250 mV at 150 µA cm^−2^, suggesting stable ion transport throughout cycling. In contrast, Pebax1657 QSPE initially exhibits a higher charge overpotential (≈900 mV at 75 µA cm^−2^ and ≈700 mV at 150 µA cm^−2^), which significantly decreases within the first ≈10 cycles, stabilizing at ≈100 mV and ≈200 mV, respectively. This decline suggests an activation process, likely due to electrolyte wetting and improved ion diffusion, which may be associated with the higher Na⁺ transference number (t_Na⁺_ ≈ 0.40 vs Celgard's 0.32). At higher current density, both systems exhibit an increase in overpotential due to polarization effects. However, Pebax1657 QSPE stabilizes at lower overpotential values than its initial state, indicating progressive improvement in electrochemical performance over cycling. These results underscore the ability of the Pebax1657‐based electrolyte to reduce charge overpotential after initial conditioning.

It can be concluded that Celgard‐assembled cells exhibit slightly better performance at lower current density (75 µA cm^−2^) compared to the Pebax1657 QSPE cells, maintaining capacity and CE over a longer period. However, at higher current densities, Pebax1657 QSPE cells demonstrate superior cycling performance, achieving 35 cycles at 150 µA cm^−2^ compared to 25 cycles at 75 µA cm^−2^ in Celgard‐based cells. Additionally, Pebax1657 QSPE exhibits a lower charge overpotential (250 vs 200 mV) and maintains a stable C.E. of ≈83% after a pre‐activation phase of ≈10 cycles. In summary, the cycling shows that the QSPE exhibits lower charge overpotentials (≈0.2 V), improving CE (80‐90%) and cycle retention (35 cycles at 150 µA cm^−2^). Thus, despite a slightly lower conductivity, the superior Na^+^ transport, stable cycling, and controlled discharge product formation ensure that the QSPE does not negatively impact rate capability or long‐term performance.

The SEM images and photos in Figure S15 (Supporting Information) illustrate the changes in Na metal anodes after the galvanostatic discharge in Na–O_2_ batteries using Pebax1657 QSPE and Celgard/L.E. at 75 µA cm^−2^. As shown in Figure S15 (Supporting Information), the pristine Na anode (Figure S15a,d, Supporting Information) displays a smooth surface, typical of uncycled Na metal. After discharge at 75 µA cm^−2^ in Na–O_2_ cells, the Na electrodes recovered from both Pebax1657 QSPE (Figure S15b,e, Supporting Information) and Celgard/L.E. (Figure S15c,f, Supporting Information) do not show dendritic structures, but rather exhibit roughened surfaces with cracks. This morphology likely results from the formation of a passivation layer and/or localized Na plating/stripping, which is common in metal‐O_2_ systems. Despite differences between the dense Pebax1657 QSPE system and the porous Celgard/L.E. system, as well as the higher Na⁺ transference number of Pebax QSPE (t_Na⁺_ ∼ 0.40) compared to Celgard/L.E. (t_Na⁺_ ∼ 0.32), both separators result in similar passivation and cracking on the Na metal anode with no evidence of dendrite formation under the tested conditions.

In Na–O_2_ batteries, capacities and current densities are typically calculated based on the mass (gravimetric) and area (areal) of the air‐cathode. To enable a meaningful comparison with existing studies on Ionogel electrolyte^[^ ^8^ ^]^ and quasi‐polymer electrolytes (PVDF‐HFP/4% SiO_2_,^[^ ^9^ ^]^ PEO/NaTFSI/25 wt.% NZSP,^[^ ^13^ ^]^ and PVB‐PDADMATFSI‐PVB copolymer,^[^ ^36^ ^]^ respectively), the performance of the cells in Table S4 (Supporting Information) was assessed using areal units.

Pebax1657 QSPE demonstrated a higher capacity (2.60 mAh cm^−2^ at 75 µA cm^−2^), outperforming other electrolyte systems reported in the literature. For instance, the ionogel electrolyte exhibited a significantly lower capacity of 0.17 mAh cm^−2^ at 25 µA cm^−2^, while the PEO/NaTFSI/25 wt.% NZSP system delivered 2.39 mAh cm^−2^ at 44 µA cm^−2^. Similarly, the PVB‐PDADMATFSI‐PVB block copolymer achieved a capacity of 1.59 mAh cm^−2^ at 75 µA cm^−2^, which remains lower than the performance observed for Pebax1657 QSPE. Moreover, this capacity was achieved at a higher cutoff potential (1.8 vs 1.6 or 1.5 V) and higher current densities than other electrolyte systems.

Among the studies that report cycling data using areal units, only two works (PEO/NaTFSI/25 wt.% NZSP^[^ ^13^ ^]^ and PVB‐PDADMATFSI‐PVB copolymer^[^ ^36^ ^]^), provide direct comparisons. Pebax1657 QSPE exhibits a significantly longer cycle life than other reported systems. This study achieved 35 cycles at 150 µA cm^−2^ with a limited capacity of 0.25 mAh cm^−2^, while PEO/NaTFSI/25 wt.% NZSP system^[^ ^13^ ^]^ delivered 25 cycles at similar capacity limitation (0.22 mAh cm^−2^) but at a lower current density (44 µA cm^−2^). Additionally, the PEO/NaTFSI/25 wt.% NZSP system operated within a larger voltage window (1.6–4.5 V) and exhibited a higher cycling overpotential (0.3–1.3 V) compared to Pebax1657 QSPE.

While the PVB‐PDADMATFSI‐PVB copolymer system^[^ ^36^ ^]^ delivered a capacity of 1.59 mAh cm^−2^ at 75 µA cm^−2^, this is lower than the capacity (2.60 mAh cm^−2^) achieved by Pebax1657 QSPE under the same current conditions. These key parameters contribute to reducing energy loss in the battery and mitigating side reactions, enhancing overall efficiency. Notably, its cycling overpotential stabilized below 0.2 V after eight cycles, indicating efficient charge transfer and reduced polarization. In terms of C.E., Pebax1657 QSPE exhibited a lower CE (≈85%) compared to other systems.

Furthermore, Table S4 (Supporting Information) shows the gravimetric energy density of different electrolyte systems for Na–O_2_ batteries, considering both active material and substrate mass. The Pebax1657 QSPE system demonstrated the highest energy density at 377.42 Wh/kg, outperforming the PEO/NaTFSI/NZSP (340.76 Wh/kg), PVB‐PDADMATFSI‐PVB (182.21 Wh/kg), and the Ionogel electrolyte (213.04 Wh/kg) systems due to their limited areal capacity. Despite these improvements, all systems remain significantly below the theoretical energy density (≈1060 Wh kg^−1^), primarily due to inactive material contributions, side reactions, and interfacial resistances. The higher energy density of Pebax1657 QSPE suggests that its reduced PEO crystallinity and formation of orderly nanodomains improved the ionic conductivity and sodium transfer number, leading to enhance battery performance. Conversely, PEO and PVB‐based systems likely suffer from higher mass contributions (e.g., polymer binders, inorganic fillers, and packed dense structure), reducing their energy output. To bridge the gap between theoretical and practical performance, future research should focus on minimizing inactive material mass, optimizing electrode‐electrolyte interfaces, and mitigating side reactions such as electrolyte decomposition and sodium dendrite formation.

However, it is important to remark that Pebax1657 QSPE exhibits a Na⁺ transference number (t_Na⁺_) of ≈0.40 and an ionic conductivity of 6.57 × 10^−4^ S cm^−1^ at room temperature (RT.), which are competitive with other Na‐ion polymer electrolytes. To better evaluate the performance of Pebax1657 QSPE, a comparison with other Na‐ion polymer electrolytes is summarized in Table S5 (Supporting Information), and some detailed information is discussed here as well. For instance, higher t_Na⁺_ is reported for PEO/PNaMTFSI^[^ ^44^ ^]^ (> 0.83) and NaPTAB‐SGPE^[^ ^45^ ^]^ (0.91), however, Pebax1657 QSPE ionic conductivity (6.57×10^−4^ S cm^−1^) at room temperature remains significantly higher than these systems, which exhibit lower ionic conductivity range (≈10^−4^–10^−5^ S cm^−1^). In contrast, PVDF‐HFP‐based GPE (PSIL70)^[^ ^46^ ^]^ achieves higher ionic conductivity (1.9×10^−3^ S cm^−1^) and lower t_Na+_ (0.27) than Pebax1657 QSPE, indicating a trade‐off between these key parameters (t_Na⁺_ and ionic conductivity). Notably, NASICON‐based fillers^[^ ^47^ ^]^ and PEO/Na_3_SbS_4_^[^ ^28^ ^]^ show slightly higher t_Na⁺_ and ionic conductivity (0.57 and 2.78×10^−3^ S cm^−1^, 0.49 and 1.33×10^−4^ S cm^−1^, respectively) compared with Pebax1657 QSPE, likely due to rigid frameworks and stronger Na⁺ coordination, which enhance cation selectivity but limit overall ion mobility.^[^ ^28^, ^47^ ^]^ Furthermore, Pebax1657 QSPE exhibits an oxidation onset potential of 4.69 V versus Na/Na^+^, which surpasses conventional PEO/PNaMTFSI‐based electrolytes^[^ ^44^ ^]^ (3.5–4.5 V) and PVDF‐HFP‐based GPEs^[^ ^46^ ^]^ (4.2 V), demonstrating superior electrochemical stability. While its stability is slightly lower than NaPTAB‐SGPE^[^ ^48^ ^]^ (5.2 V) and PVDF‐HFP/PMMA‐based GPE^[^ ^49^ ^]^ (5.0 V), it remains well‐suited for Na–O_2_ battery applications. Furthermore, Pebax1657 QSPE enables Na–O_2_ cells with lower overpotentials (≈0.2 V) and high CE (80‐90%), reinforcing its potential as a competitive electrolyte. These results highlight that while further optimization could enhance its performance, the developed Pebax1657 QSPE already presents a well‐balanced combination of high conductivity, moderate (t_Na+_), and robust electrochemical stability, making it a promising candidate for Na–O_2_ and Na‐ion battery applications.

## 3 Conclusion

In this study, a novel QSPE based on the Pebax1657 copolymer with 1 M NaTFSI in diglyme was developed for quasi‐solid‐state Na–O_2_ batteries. Compared to the conventional liquid electrolyte (Celgard/L.E.), Pebax1657 QSPE showed improved electrochemical (≈390 mV) and thermal properties, likely due to chemical interactions between the electrolyte and the functional groups of Pebax1657. Structural analysis (Raman spectroscopy, DSC, XRD, and SAXS) revealed reduced crystallinity of the copolymer and the formation of orderly nanodomains upon electrolyte incorporation, which can lead to enhanced Na^+^ transport. This hypothesis was corroborated by the higher Na^+^ transference number (t_Na⁺_ ∼ 0.40) compared to conventional liquid electrolyte using a Celgard separator (t_Na⁺_ ∼ 0.32) and PEO‐based QSPE (t_Na⁺_ < 0.25), attributed to strong hydrogen bonding between PA and PEO segments. While the Pebax1657 membrane exhibited slightly higher electrolyte uptake (50.78%) than Celgard (45.39%), the porous structure of Celgard resulted in marginally higher ionic conductivity (7.24 × 10^−4^ S cm^−1^ vs 6.57 × 10^−4^ S cm^−1^), despite the strong electrostatic interactions and dense structure of Pebax1657.

Galvanostatic cycling in Na|Na symmetrical cells demonstrated Pebax1657 QSPE's superior stability, maintaining low overpotentials (≈80 mV) for 210 h at 150 µA cm^−2^, whereas Celgard/L.E. exhibited a rapid overpotential increase to 700 mV after 110 h, likely due to free electrolyte which is prone to electrochemical degradation and/or Na interfacial degradation.

In full Na–O_2_ discharge tests, Pebax1657 QSPE produced smaller, more uniform NaO_2_ particles, improving cathode utilization and reducing passivation effects. Additionally, Pebax1657 QSPE exhibited lower charge overpotentials, which enhanced CE during deep cycling, likely due to the morphology of the discharge products. While Celgard/L.E. displayed better initial stability at 75 µA cm^−2^ (40 cycles, ≈90% C.E.), its performance declined after 20 cycles at 150 µA cm^−2^. In contrast, Pebax1657 QSPE extended cycle life to 35 cycles at 150 µA cm^−2^, maintaining a stable C.E. (≈85%) after initial activation.

In summary, Pebax1657 QSPE presents a promising alternative for quasi‐solid‐state Na–O_2_ batteries, offering enhanced safety, efficiency, and durability. It exhibits well‐balanced electrochemical performance, with high‐capacity retention at elevated current densities and lower overpotentials, outperforming commercial Celgard/L.E. systems by enabling higher discharge rates and improved efficiency. Its stable cycling and enhanced ion transport underscore the importance of QSPE materials in advancing Na–O_2_ battery technology.

However, further optimization is required to improve CE and extend cycle life. Future research should focus on electrolyte design and interfacial stability to maximize long‐term performance and facilitate the practical implementation of Pebax1657‐based systems. These findings highlight the potential of advanced polymer electrolytes in driving innovation toward solid and quasi‐solid‐state energy storage technologies.

## 4 Experimental Section

### Synthesis of Pebax1657 Membrane

The Pebax1657 polymer was provided as pellets by Arkema (France) and absolute ethanol (EtOH, purity > 99.5%) was purchased from Sigma–Aldrich. The Pebax1657 membrane was prepared, as depicted in Figure S1 (Supporting Information), using solvent evaporation casting method as previously reported.^[^ ^15b,d^ ^]^ Polymer solutions of 3 wt.% in a 70:30 EtOH/H_2_O mixture were stirred at 80 °C under reflux at 500 rpm. The polymer was gradually dissolved to avoid agglomeration formation. The resulting solution was poured into a glass Petri dish, covered, and allowed to evaporate at room temperature for 48 h, followed by vacuum drying at 60 °C overnight. The dried membrane was then carefully peeled off from the glass dish for subsequent physicochemical and electrochemical testing. The fabricated Pebax1657 membrane was characterized and compared to Celgard H2010, a polymer widely used as a Na–O_2_ battery separator. Celgard separator was included in this study as reference to compare common liquid electrolytes (separator/liquid electrolyte). For simplicity, the Pebax1657 membrane and Celgard separator in contact with the liquid electrolyte (1 M NaTFSI in diglyme) will be referred to as Pebax1657 QSPE and Celgard/L.E., respectively.

### Physicochemical Characterizations of the Membranes

The chemical stability of the pristine Pebax1657 membrane in contact with the liquid electrolyte (1 M NaTFSI in diglyme) was studied via liquid‐NMR. The experiments were performed at 30 °C on a 300 Bruker Avance III HD spectrometer under a TopSpin 3.5 and equipped with a Bruker BBFO z‐gradient 5 mm probe head. A small piece of the Pebax1657 membrane was immersed for 48 h in the electrolyte inside the NMR tube, with deuterated DMSO as a reference. 1H spectra were acquired with 128 transients, a spectral width of 6009.6 Hz, a recycle delay of 1.0 s, an acquisition time of 5.45 s, a 90‐flip angle pulse of 14 µs, and 64 K data points.

The nitrogen adsorption isotherms were conducted to measure the porosity of pristine Pebax1657 membrane and Celgard. The measurements were performed at ‐196 °C using a Micromeritics ASAP 2020 instrument for relative pressures (P/P0) between ≈10^−8^ and 0.995. Samples were preliminarily outgassed for 24 h at 120 °C. The specific surface area was calculated by applying BET to the obtained isotherms. The total pore volume was calculated by the amount of adsorbed gas at (P/P_o_ > 0.8).

SEM measurements were conducted using a FESEM Apreo‐2S HiVac (ThermoFisher) to analyze the structure morphology of pristine polymers (Pebax1657 and Celgard) and Pebax1657 QSPE. The Trinity in‐lens system enabled ultra‐high‐resolution imaging, while the integrated EDS facilitated elemental analysis. Additionally, the instrument features ColorSEM technology, which provides colorized images that combine structural details with compositional data, enabling visualization of the electrolyte distribution within Pebax1657 QSPE. To prevent exposure to ambient air, the samples were transferred to the FESEM chamber using a hermetic transfer system.

TGA technique was used to measure the thermal stability and composition of the samples (Pebax1657 and Pebax1657 QSPE). The measurements were carried out using a thermogravimetric analyser (TG 209 F1 Libra, Netzsch), 5 mg of the samples placed in an alumina crucible with a volume of 85 µL at a constant heating rate of 10 °C min^−1^ and 60 mL min^−1^ of synthetic argon.

DSC (Q2000, TA Instruments) was employed to examine the phase transition behavior of the samples (Pebax1657 and Pebax1657 QSPE). The samples, weighing ≈5–10 mg, were sealed in aluminium crucibles under argon in a glove box. Each sample underwent two consecutive scans with cooling and heating rates of 10 °C min^−1^ over a temperature range of −80–250 °C. The glass transition temperature (Tg, indicating the onset of heat capacity change) and melting temperature (Tm, corresponding to the peak of the endothermic reaction) were determined from the resulting curves.

FTIR measurements were conducted to identify the chemical bonds and structure for the samples (Pebax1657, Celgard, Pebax1657 QSPE, and Celgard/L.E.). The measurements were performed using flexible Benchtop FTIR Spectrometer Agilent Cary 630 FTIR spectrometer inside the glovebox connected with a DTGS detector and an ATR diamond accessory with a wavenumber resolution ≤ 2 cm^−1^ and measured in the range 4000–600 cm^−1^.

XRD measurements were used to determine the crystalline structure of the materials (Pebax1657, Celgard, Pebax1657 QSPE, and Celgard/L.E.). The measurements were conducted using a Bruker DISCOVER system with a Cu‐Kα X‐ray tube and a LYNXEYE XE detector, and a 2θ active length of 3.79°. Each sample was scanned in the Bragg‐Brentano geometry, covering a scattering angle range of 5°–120° in 0.1° increments, with an acquisition time of 6.25 s per step.

SAXS was conducted to analyze the nanoscale internal structure and organization of the materials (Pebax1657, Celgard, Pebax1657 QSPE, Celgard/L.E.). The measurements were performed using a Bruker Nanostar instrument with a CuK‐α source (λ ≈ 1.542 Å) operating at 40 mA and 40 kV. The setup included three pinhole collimators, an evacuated beam path, and a Vantec 2000 2D detector (14 mm × 14 mm, 2048 × 2048 pixels). A nickel foil filtered out Kβ radiation. The sample‐to‐detector distance was 108 cm, covering a momentum transfer range of 0.01 < q < 0.2 Å^−1^. Membrane samples (4 mm diameter) were mounted on a steel holder and exposed for 1 h. Intensity measurements were corrected for sample transmission and holder scattering was subtracted. The q values for Pebax scattering peaks were determined using SasView, and the Bragg distance (d‐spacing) was calculated with d = 2π/q.

The electrolyte uptake (%) was determined by immersing 13 mm diameter discs of the pristine polymers (Pebax1657 and Celgard) in 1 mL electrolyte solution (1 M NaTFSI in diglyme). After 1 h, the membranes were removed, and excess liquid was eliminated using filter paper. The weight gain of the membrane was then measured. Subsequently, the membrane was re‐immersed in the electrolyte, and the process was repeated after a specified period (1–6 h.) to assess the absorption capacity following Equation (1):

(1) $$EU\left(\%\right)=\frac{W_{f}-W_{0}}{W_{0}}x100$$

where EU stands for electrolyte uptake, W_f_ final weight and W_o_ initial weight of the polymer materials.

The Gurley porosity measurements were conducted using a Gurley densometer (GENUINE Gurley, 4320 automatic Digital Timer) to evaluate the air permeability and porosity of the pristine membranes (Pebax1657 and Celgard). The recorded number corresponds to the time required for a specific amount of air to pass through the membrane under a specific pressure. The standardized measurements were performed by using the 1 in 2 (2.54 cm^2^) plates and 100 cm^3^ of air.

### Postmortem Analysis

Raman spectroscopy measurements were performed to determine the chemical nature of the discharge products formed on the surface of the different cathodes at different current densities (75 and 150 µA cm^−2^) using a Renishaw Raman spectrometer (Nanonics Multiview 2000) operating with an excitation wavelength of 532 nm. The spectra were obtained by performing 5 acquisitions with 30 s of dwell time at 0 ppm. Before measurements, the different cathodes were washed with ethylene glycol dimethyl ether (DME) to remove the excess of salt, dried and transferred to the different characterization equipment using air‐tight holders to avoid the degradation of the air/moisture sensitive products.

In order to evaluate the discharge product morphology after electrochemical tests, the discharged cathodes were washed in the glovebox using dried DME prior to the SEM imaging. The electrodes were transferred to the SEM chamber using a hermetic transfer chamber to avoid exposure to ambient air. SEM measurements were performed using a series microscope and a FEI Quanta 250 microscope operating at 20 kV. The micrographs from JSM IT 300 were obtained in secondary electron mode, with a 5 kV accelerating voltage, 30 nA probe current. To get more detail of the discharge products homogeneity and distribution on the electrode surface, the SEM micrographs (FEI‐Quanta 200 FEG) were obtained using the secondary electron detector (ETD), with a 10 kV accelerating voltage and a value for the spot size of 3.

### Full Na–O_2_ Battery Assembly and Testing

Ionic conductivity tests were made on SAS VMP3 potentiostat (Biologic) using CR2032 coin cells assembled in an argon‐filled glove box. The ionic conductivity of the Celgard/L.E. and the Pebax1657 QSPE was measured using alternating‐current (AC) impedance spectroscopy over a frequency range of 10^4^ to 10^−1^ Hz at room temperature. Two stainless steel (SS) disks were used as current collector electrodes in a SS|Celgard/L.E.|SS; SS| Pebax1657 QSPE |SS configurations. The ionic conductivity (σ) was calculated using Equation (2):

(2) $$\sigma =\frac{1}{R}\left(\frac{l}{A}\right)$$

where l is the membrane thickness (cm), A is the active surface area (cm^2^), and R is the total resistance (Ω). For the Electrochemical impedance spectroscopy (EIS) measurements, a coin cell with the electrolyte, having a 14 mm diameter and 40 µm thickness, was employed.

Plating‐striping tests were made on SAS VMP3 potentiostat (Biologic) using CR2032 coin cells assembled in an argon‐filled glove box. The measurements were conducted on symmetrical Na|Celgard/L.E.|Na; Na| Pebax1657 QSPE |Na configurations (12 mm sodium chips Xiamen AOT battery equipment Technology Co.) to examine the electrochemical stability of the electrolyte/Na electrode interface and dendrite formation. Two different measurements were conducted: first, an open‐circuit voltage (OCV) measurement was performed for 30 min; second, the cells were cycled at varying current densities from 25 to 100 µA cm^−2^ for 5 cycles, each half‐cycle in these measurements was performed for 1 hr.

LSV experiment was conducted to assess the electrochemical stability of the Celgard/L.E. and the Pebax1657 QSPE electrolytes using an unsymmetrical coin cell (Na|Celgard/L.E.|Na; Na| Pebax1657 QSPE |Na) with a sodium metal electrode and a stainless‐steel counter electrode. The current density was swept from (0 to 100 µA cm^−2^) within 3.0 to 5.5 V potential range at a scan rate of 1 mV s^−1^. This slow scan rate minimized capacitive currents, allowing for accurate detection of electrochemical reactions and providing a clear measure of the electrolyte's stability and breakdown voltage.

The Na^+^ transference number (t_Na₊_) was determined using two electrochemical techniques: Potentiostatic Electrochemical Impedance Spectroscopy (PEIS) and Chronoamperometry (CA), performed simultaneously in symmetrical coin cells. PEIS measured impedance across a frequency range from 1 MHz to 100 mHz, providing insights into ionic and electronic resistances essential for calculating the transference number. CA involved a 30‐min potential step at 10 mV to analyze the current–time response and concentration gradients. The (t_Na₊_) was calculated using the Bruce and Vincent equation,^[^ ^18^ ^]^ as presented in Equation 3, which relates initial and steady‐state currents (I_0_ and I_ss_) from CA and initial and final interfacial resistances (R_io_ and R_iss_) from PEIS under a 10 mV applied potential difference.

(3) $$t_{Na^{+}}=\frac{I_{ss}\left(\Delta V-I_{o}R_{i,o}\right)}{I_{o}\left(\Delta V-I_{ss}R_{i,ss}\right)}$$

Swagelok‐type cells modified with an oxygen reservoir were used to conduct battery testing. The cells were dried for 24 h at 60 °C in vacuum, prior to their transference to an argon filled glovebox (H_2_O < 0.1 ppm, O_2_ < 0.1 ppm, Jacomex). Sodium metal discs of (12 mm, Panreac) and Freudenberg H23C6 carbon paper (12 mm, Quintech) were used as anode and air‐cathode, respectively. The carbon paper was dried overnight at 200 °C in vacuum and supported in a 12 mm diameter stainless steel mesh (Alfa Aesar), acting as porous current collector. A 1 M NaTFSI in DEGDME, (anhydrous, 99.5% Sigma Aldrich) was used as electrolyte. DEGDME was dried over molecular sieves (3 Å, Sigma Aldrich) for one week. The sodium salt (NaTFSI, 99.5%, Solvionic) was dried under vacuum at 110 °C for 48 h. The water content in the electrolyte was measured with an 899 Karl Fisher Coulometer (Metrohm) and was below 10 ppm. Finally, commercial Celgard H2010 and the fabricated Pebax1657 membranes were tested as QSPE. They were cut in circles of 13 mm, dried in a Buchi oven at 100 °C for 24 h, and soaked in 200 µL (1 M NaTFSI in diglyme) prior to the cell assembly. The thus prepared Na–O_2_ cells were pressurized with pure oxygen (99.99% pure) to ≈1 atm and rested for 8 h at open circuit voltage ≈ 2.2–2.3 V (vs Na^+^/Na) before the electrochemical measurements. Galvanostatic charge‐charge experiments were performed using a SAS VMP3 potentiostat (Biologic) at a current density of 75 and 150 µA cm^−2^. The cells assembled using the different QSPEs were deep discharged to 1.8 V. Cycle life assessment was conducted by full discharge of the battery from 1.8 to 3.2 V (vs Na^+^/ Na) or by shallow cycling at a capacity cut‐off of 0.25 mAh cm^−2^.

## Conflict of Interest

The authors declare no conflict of interest.

## Supporting information



Supporting Information

## Data Availability

The data that support the findings of this study are available from the corresponding author upon reasonable request.
