# Supplementary material for: A New Quasi‐Solid Polymer Electrolyte for Next‐Generation Na–O2 Batteries: Unveiling the Potential of a Polyamide‐Polyether System
Source: Adv Sci (Weinh). 2025 May 23;12(30):e04490. doi: 10.1002/advs.202504490 (PMC12376506; doi:10.1002/advs.202504490)
Supplement: Supplementary file 1 — Supporting Information [file ADVS-12-e04490-s001.docx]

**Supporting Information**

A new Quasi-solid Polymer Electrolytes for Next-Generation Na-O_2_ Batteries: Unveiling the potential of a Polyamide-Polyether System

Mohamed Yahia^a^, Marina Enterría^a^, Cristina Pozo-Gonzalo ^b,c,*^, Nagore Ortiz-Vitoriano^a,d,*^

*^a^ Center for Cooperative Research on Alternative Energies (CIC energiGUNE), Basque Research and Technology Alliance (BRTA), Alava Technology Park, Albert Einstein 48, 01510, Spain*

*^b^ Institute for Frontier Materials, Deakin University, Geelong, Victoria, 3200, Australia*

*^c^ Instituto de Carboquímica (ICB-CSIC), C/Miguel Luesma Castán, 4, 50018, Zaragoza, Spain*

*^d^ Ikerbasque, Basque Foundation for Science, María Díaz de Haro 3, 48013 Bilbao, Spain*

^*^Corresponding authors: Nagore Ortiz-Vitoriano ([nortiz@cicenergigune.com](mailto:nortiz@cicenergigune.com)) and Cristina Pozo-Gonzalo ([cpozo@csic.es](mailto:cpozo@csic.es))

**INDEX**

**Figures**

**Figure S1.** The synthetic route for the Pebax®1657 membrane via solvent evaporation method.

**Figure S2.** SEM micrographs corresponding of the Celgard separator (a) and Pebax®1657 membrane (b), air permeability assessed via Gurley measurements (c), N_2_ adsorption-desorption isotherm at -196 ºC (77K) (d).

**Figure S3** Electrolyte uptake of the Celgard separator and Pebax®1657 membrane as a function of time (a), Nyquist plots (b), and the correlation between electrolyte uptake and ionic conductivity for Celgard/L.E. and Pebax®1657 QSPE (c). Both polymers were immersed in 1M NaTFSI/diglyme electrolyte.

**Figure S4.** NMR spectra of the 1M NaTFSI/diglyme electrolyte after immersion of the Pebax®1657 membrane for 48 hours, compared to NaTFSI/diglyme control and using deuterated dimethyl sulfoxide (DMSO-d6) as the reference solvent: (a) 1H-NMR; (b) 23Na-NMR; (c) 19F-NMR.

F**igure S5.** Raman spectra of NaTFSI/diglyme electrolyte, Pebax®1657 membrane, and Pebax Pebax®1657 QSPE, and Pebax®1657 QSPE after cycling showing different regions of the spectrum. The Pebax®1657 membrane was immersed in 1M NaTFSI/diglyme and dried before analysis.

**Figure S6.** The proposed mechanism for Na^+^ ion transport via Pebax®1657 QSPE

**Figure S7**. ATR-FTIR spectra (a) and XRD patterns (b) of Celgard separator, NaTFSI/diglyme, and Celgard/L.E.

**Figure S8.** The correct XRD patterns and the Full Width at Half Maximum (FWHM) via the Pseudo-Voigt function for Pebax®1657 membrane and Pebax®1657 QSPE impregnated with NaTFSI/diglyme.

**Figure S9.** SEM cross-section of Pebax®1657 QSPE (a) and EDS-SEM mapping for the Pebax®1657 QSPE showing the distribution of Na, F, and S elements within the membrane matrix (b and c).

**Transference number calculations**

**Figure S10.** LSV profiles using unsymmetrical coin cells (Na| Celgard/L.E.|SS; Na| The Pebax®1657 QSPE |SS) with scan rate 1 mV/s, (a), Nyquist plots before and after polarization, (b), and chronoamperometry curves (CA) for Celgard/L.E. and Pebax®1657 QSPE with PEIS measurements (c).

**Figure S11.** Voltage profiles of Na|Na symmetrical cells using Celgard/L.E (a and c) Pebax®1657 QSPE (b and f) for different stags long-term galvanostatic cycling at current density (75 µA cm-2), respectively.

**Figure S12.** Raman spectra of the air cathodes discharged at a-b)75 µA cm^-2^ and c-d) 150 µA cm^-2^, for the Swagelok-type Na-O_2_ batteries using a-c) Celgard/L.E. and b-d) Pebax®1657 QSPE.

**Figure 13.** SEM imaging of the cathode/electrolyte interface after discharge at different current densities; a-b)75 µA cm^-2^ and c-d) 150 µA cm^-2^, for the batteries assembled using a) Celgard/L.E. and Pebax®1657 QPE systems.

**Figure S14.** Galvanostatic discharge/charge (shallow cyclability) curves at current densities (75 µA cm^-2^ and 150 µA cm^-2^) for the Swagelok-type Na-O_2_ batteries assembled cells using Celgard/L.E. (a and c) and Pebax®1657 QSPE (b and d) to a limited capacity (0.25 mAh cm^-2^) and Cut-off potential (1.8 V). The charge overpotential evolution during cycling for both Celgard/L.E. and Pebax®1657 QSPE at the two current densities (75 μA cm^-2^ and 150 μA cm^-2^) (e). Both polymers were immersed in 1 M NaTFSI/diglyme inside the glovebox.

**Figure S15.** SEM imaging and digital photos of the pristine Na anode (a, d) and Na anode for the discharged Swagelok-type Na-O_2_ batteries assembled cells at current density (75 µA cm^-2^) using Pebax®1657 QSPE (b, e) and Celgard/L.E. (c, f).

**Tables**

**Table S1.** The assigned chemical-shifts for the liquid electrolyte (NaTFSI/diglyme) as observed in the NMR spectra.

**Table S2.** The assigned FTIR bands of the Pebax®1657 QSPE, Pebax®1657 membrane, NaTFSI/diglyme electrolyte, Celgard membrane, and Celgard/L.E, respectively.

**Table S3.** The assigned Raman bands of the NaTFSI/diglyme electrolyte, Pebax®1657 membrane, Pebax®1657 QSPE, and Pebax®1657 QSPE, respectively.

**Table S4.** Comparison of the electrochemical performance of this work with reported polymer electrolytes for Na-O_2_ batteries in the literature.

**Table S5.** Comparison of the electrochemical performance of this work with reported polymer electrolytes for Na ion batteries in the literature.

**References**

**
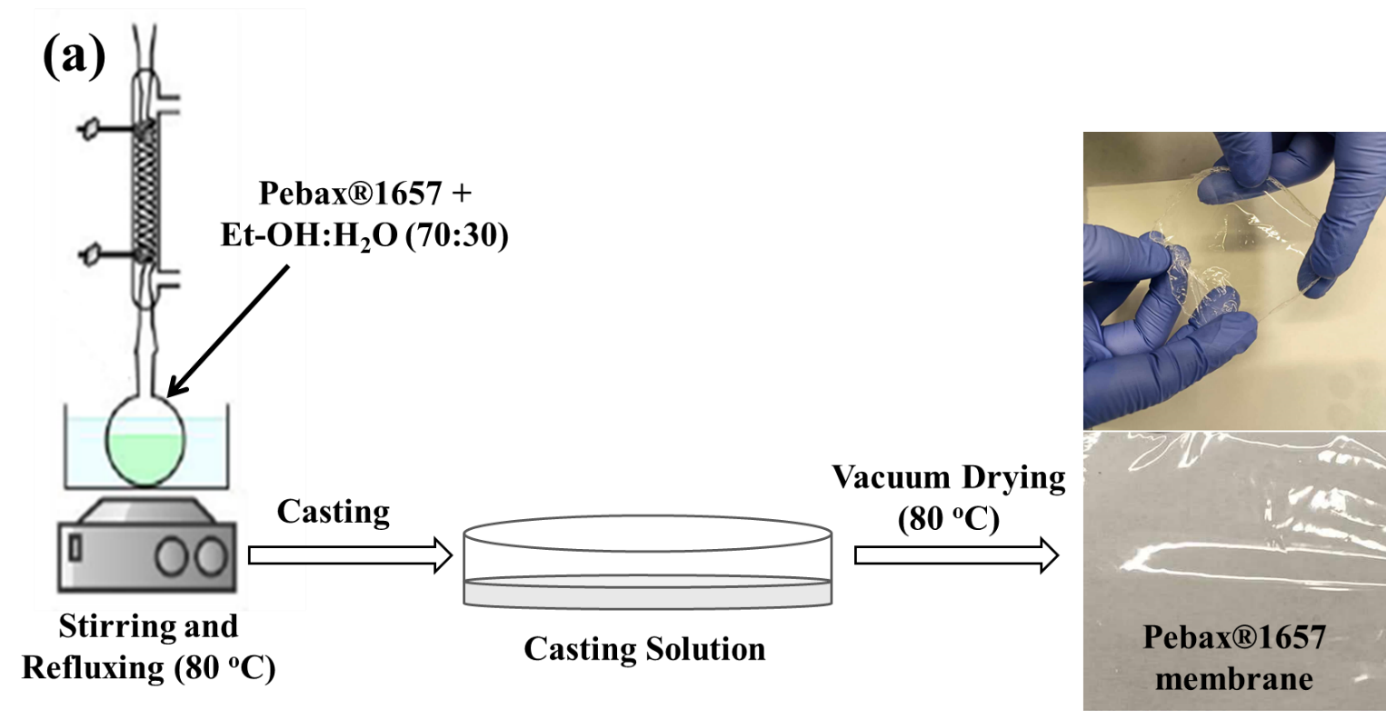
**

**Figure S1.** The synthetic route for the Pebax®1657 membrane via solvent evaporation method.

**
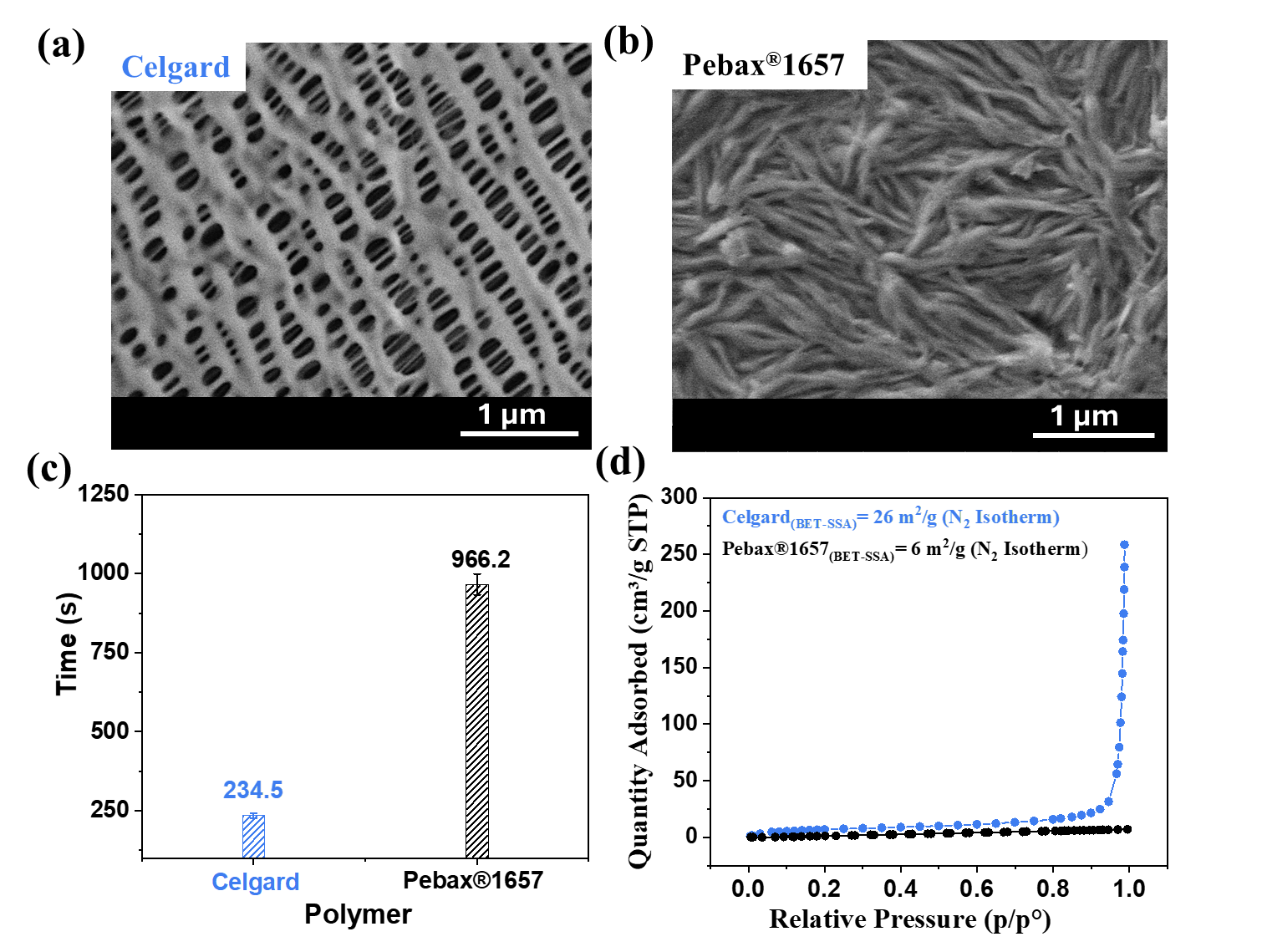
**

**Figure S2.** SEM micrographs corresponding of the Celgard separator (a) and Pebax®1657 membrane (b), air permeability assessed via Gurley measurements (c), N_2_ adsorption-desorption isotherm at -196 ºC (77K) (d).


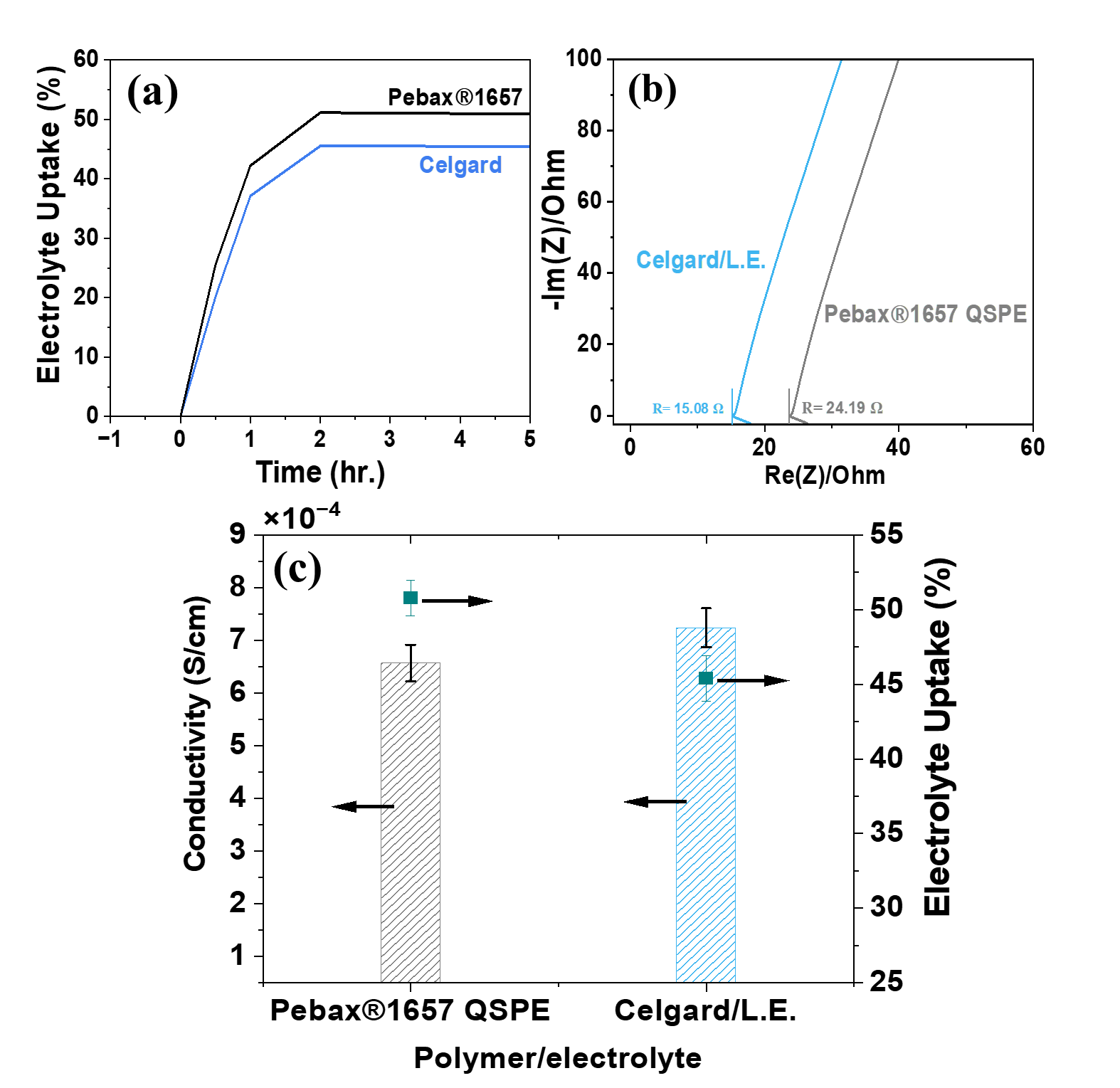


**Figure S3** Electrolyte uptake of the Celgard separator and Pebax®1657 membrane as a function of time (a), Nyquist plots (b), and the correlation between electrolyte uptake and ionic conductivity for Celgard/L.E. and Pebax®1657 QSPE (c). Both polymers were immersed in 1M NaTFSI/diglyme electrolyte.


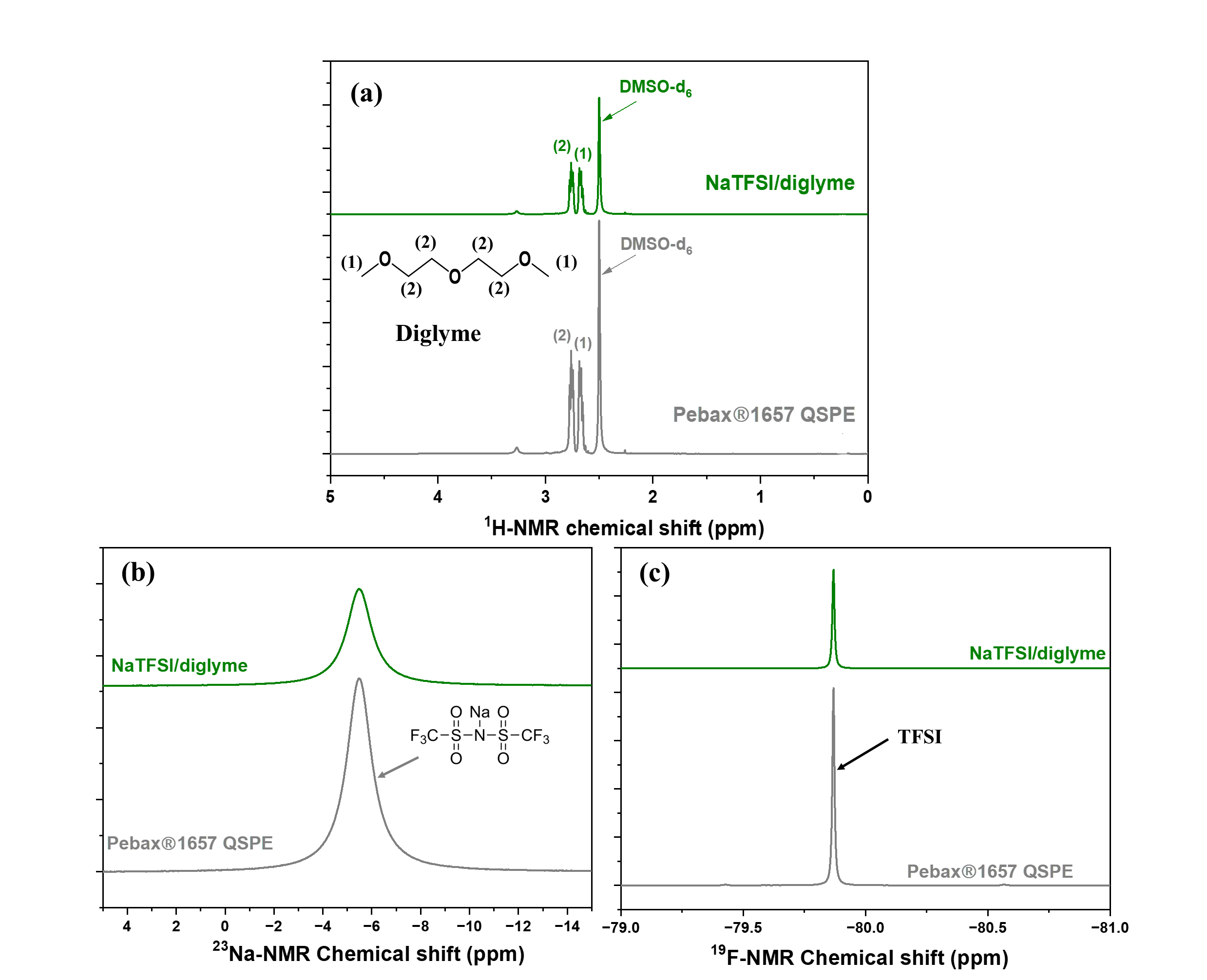


**Figure S4.** NMR spectra of the 1M NaTFSI/diglyme electrolyte after immersion of the Pebax®1657 membrane for 48 hours, compared to NaTFSI/diglyme control and using deuterated dimethyl sulfoxide (DMSO-d6) as the reference solvent: (a) ^1^H-NMR; (b) ^23^Na-NMR; (c) ^19^F-NMR.


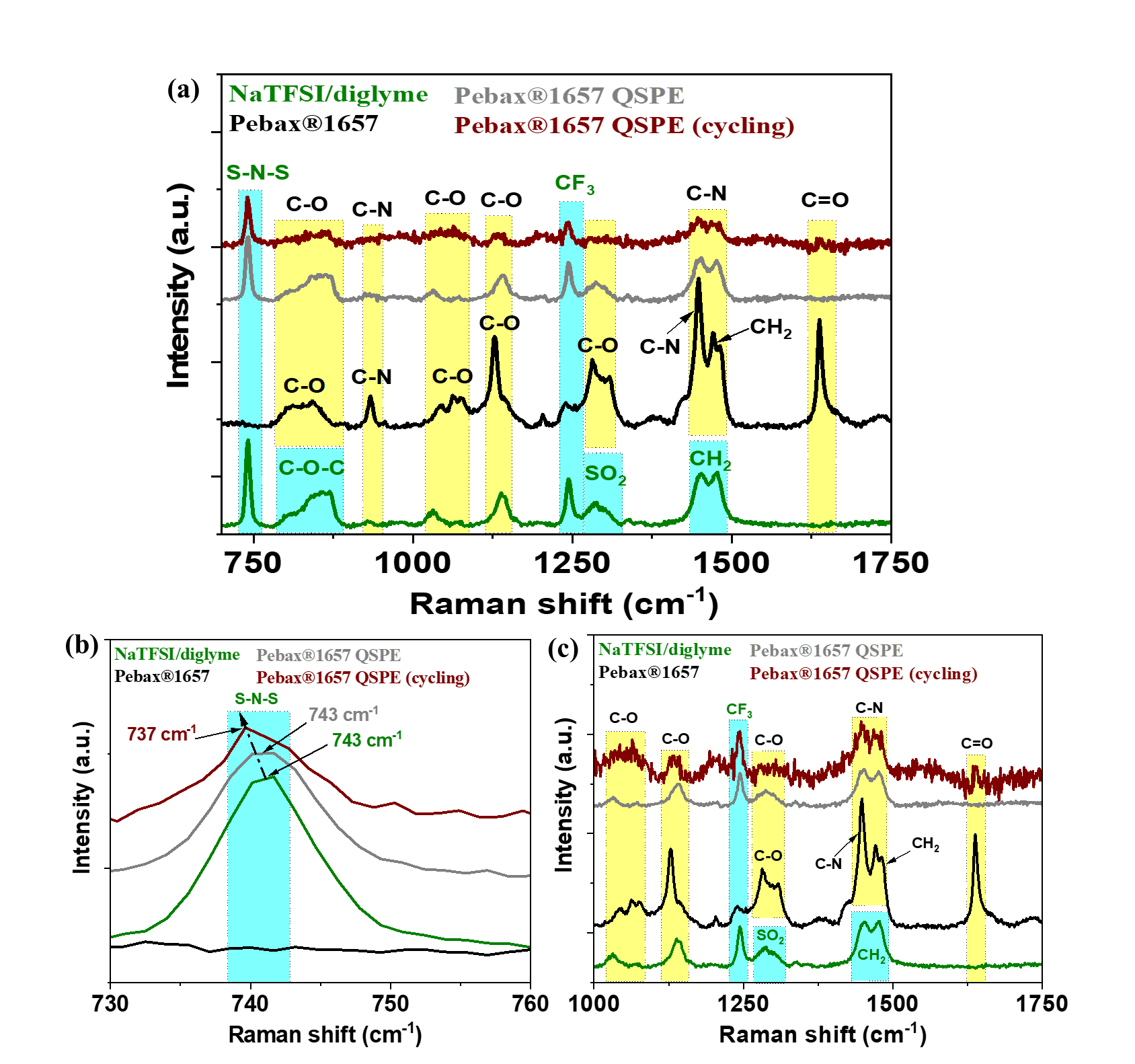


**Figure S5.** Raman spectra of NaTFSI/diglyme electrolyte, Pebax®1657 membrane, and Pebax Pebax®1657 QSPE, and Pebax®1657 QSPE after cycling showing different regions of the spectrum. The Pebax®1657 membrane was immersed in 1M NaTFSI/diglyme and dried before analysis.

**
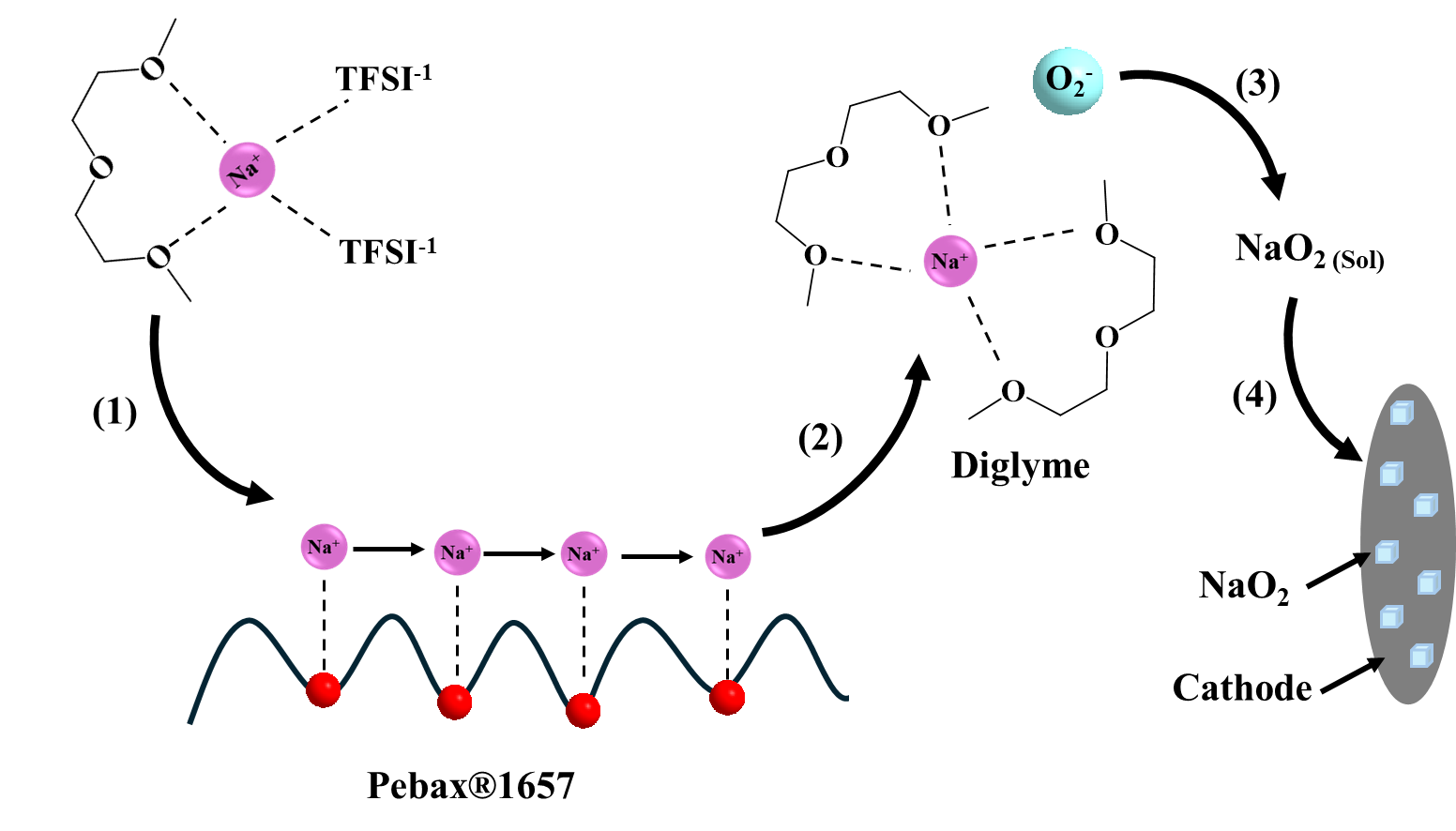
**

**Figure S6.** The proposed mechanism for Na^+^ ion transport via Pebax®1657 QSPE

**
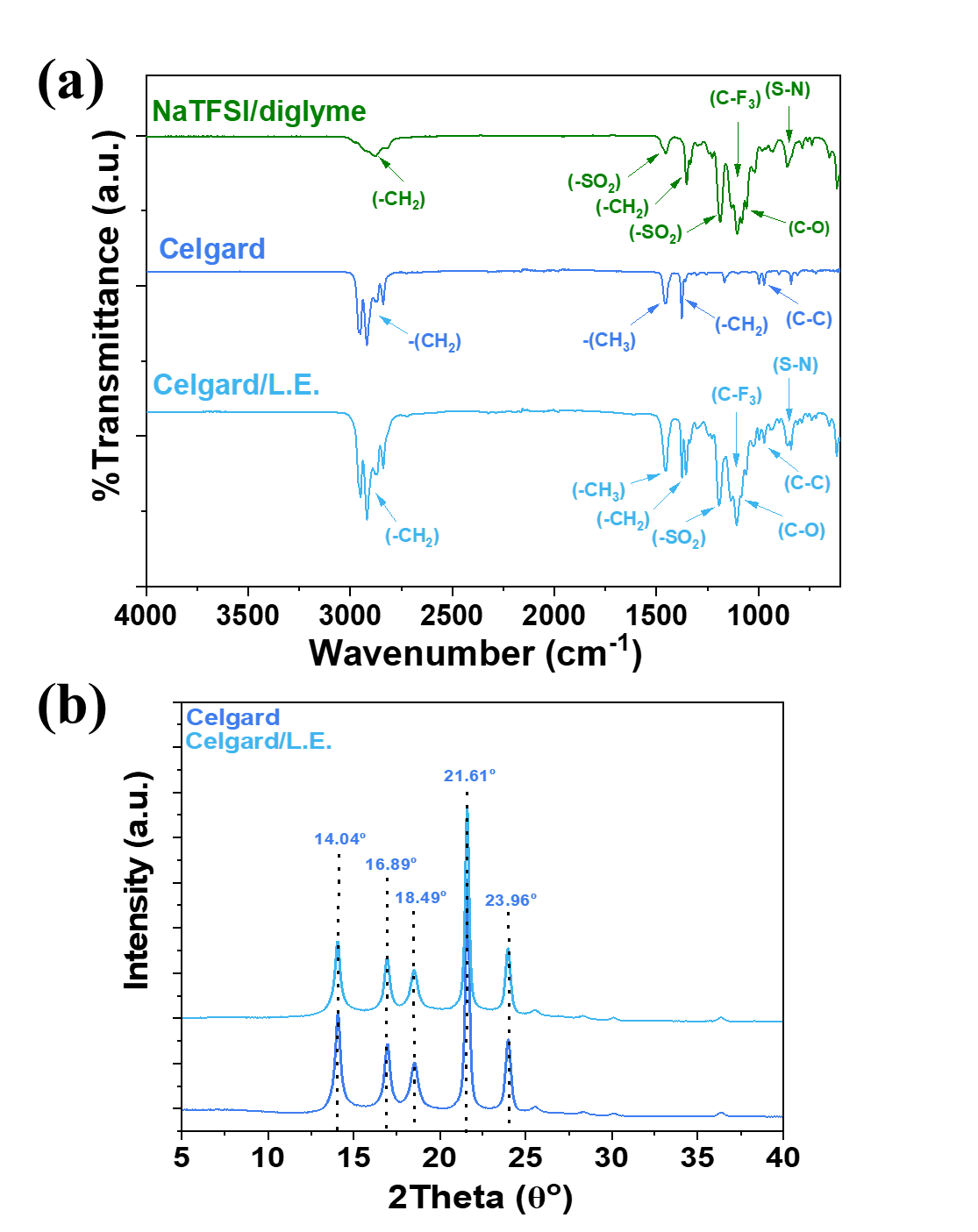
**

**Figure S7.** ATR-FTIR spectra (a) and XRD patterns (b) of Celgard separator, NaTFSI/diglyme, and Celgard/L.E.


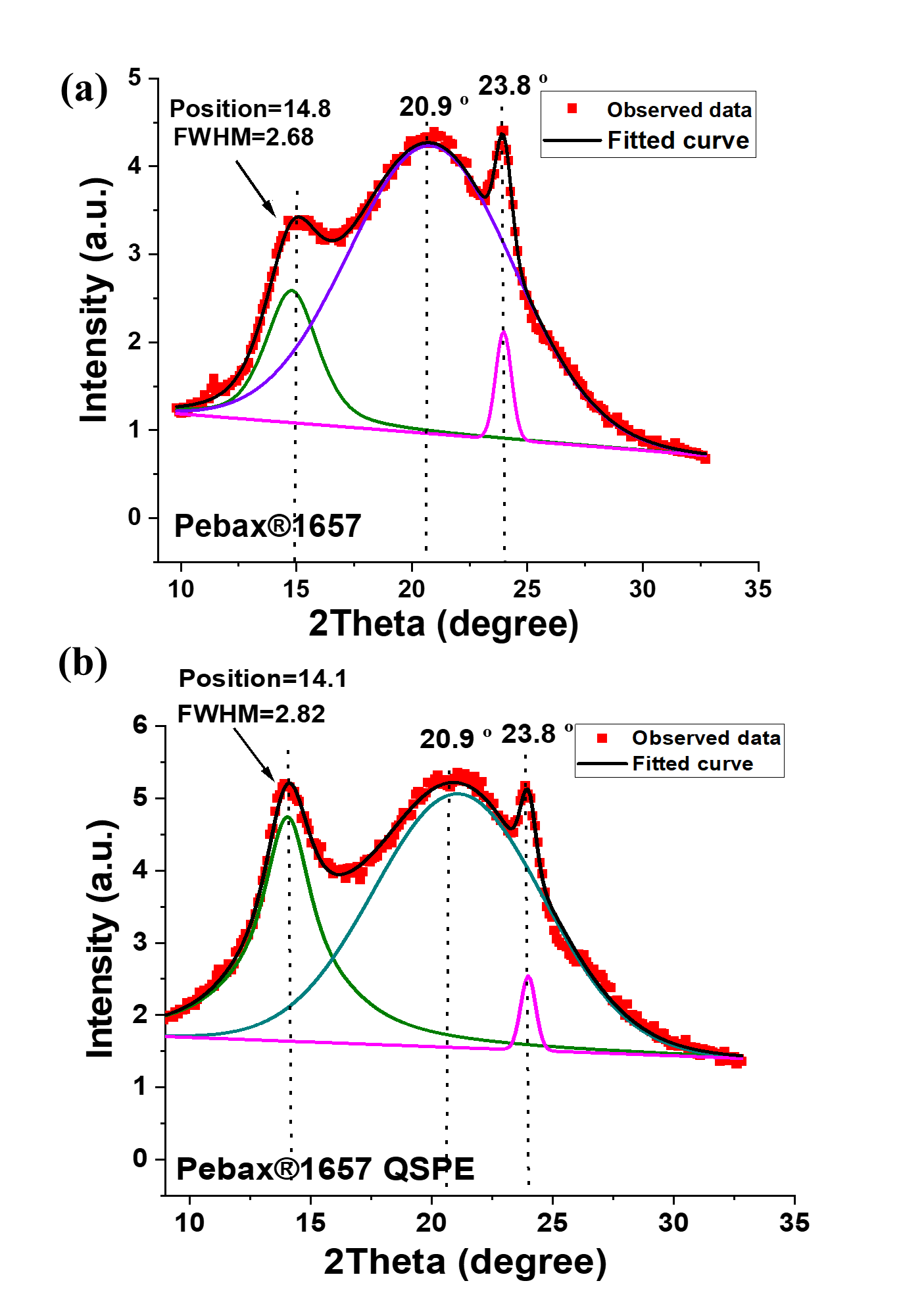


**Figure S8.** The correct XRD patterns and the Full Width at Half Maximum (FWHM) via the Pseudo-Voigt function for (a) Pebax®1657 membrane and (b) Pebax®1657 QSPE impregnated with NaTFSI/diglyme.


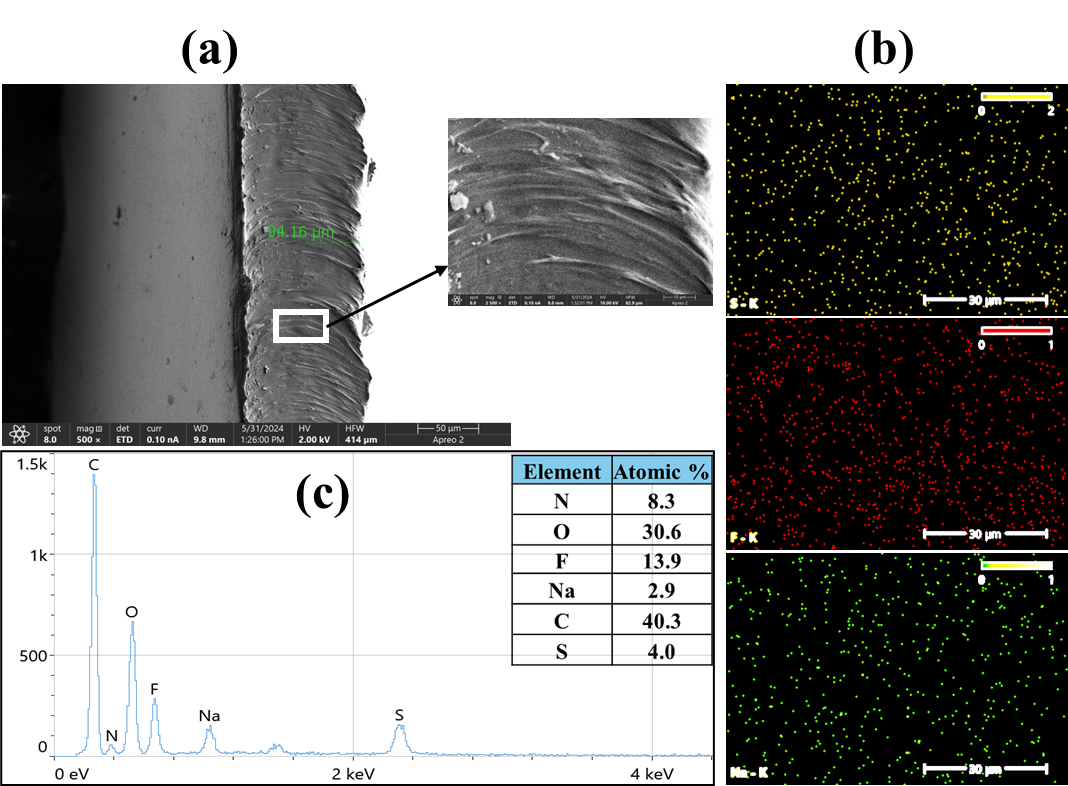


**Figure S9**. SEM cross-section of Pebax®1657 QSPE (a) and EDS-SEM mapping for the Pebax®1657 QSPE showing the distribution of Na, F, and S elements within the membrane matrix (b and c).

**Transference number calculations**

The Pebax®1657 QSPE displays slightly lower initial and final interfacial resistances (Rᵢₒ and Rᵢₛₛ) compared to Celgard/L.E., indicating a reduced charge-transfer resistance, which suggests enhanced ion conductivity in Pebax®1657. The low intercept of the Pebax®1657 plot occurs at a lower real impedance value (R₀ ≈ 20.8 Ω initially, rising to 26.2 Ω at steady state), showing a more facile initiation of ion transport compared to Celgard, which starts at a higher R₀ of 62.4 Ω initially and rises to 90.5 Ω in steady state. The high intercept of the Pebax®1657 system also remains lower than Celgard at both initial (539.4 Ω) and steady states (568.2 Ω), further supporting a reduced charge-transfer resistance in Pebax®1657 compared to Celgard, which has values of 555.8 Ω and 572.1 Ω, respectively.


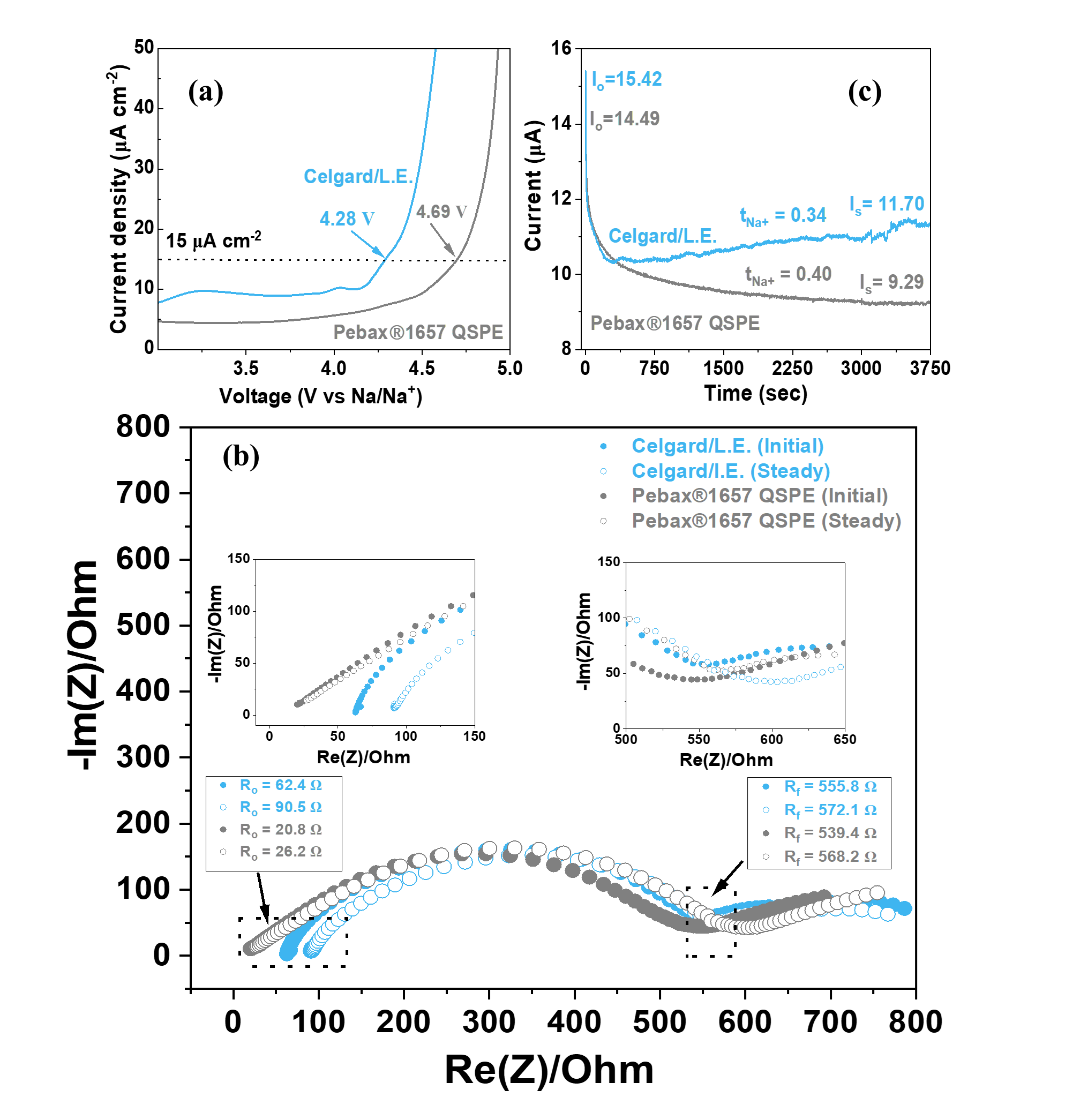


**Figure S10.** LSV profiles using unsymmetrical coin cells (Na| Celgard/L.E.|SS; Na| The Pebax®1657 QSPE |SS) with scan rate 1 mV/s, (a), Nyquist plots before and after polarization, (b), and chronoamperometry curves (CA) for Celgard/L.E. and Pebax®1657 QSPE with PEIS measurements (c).


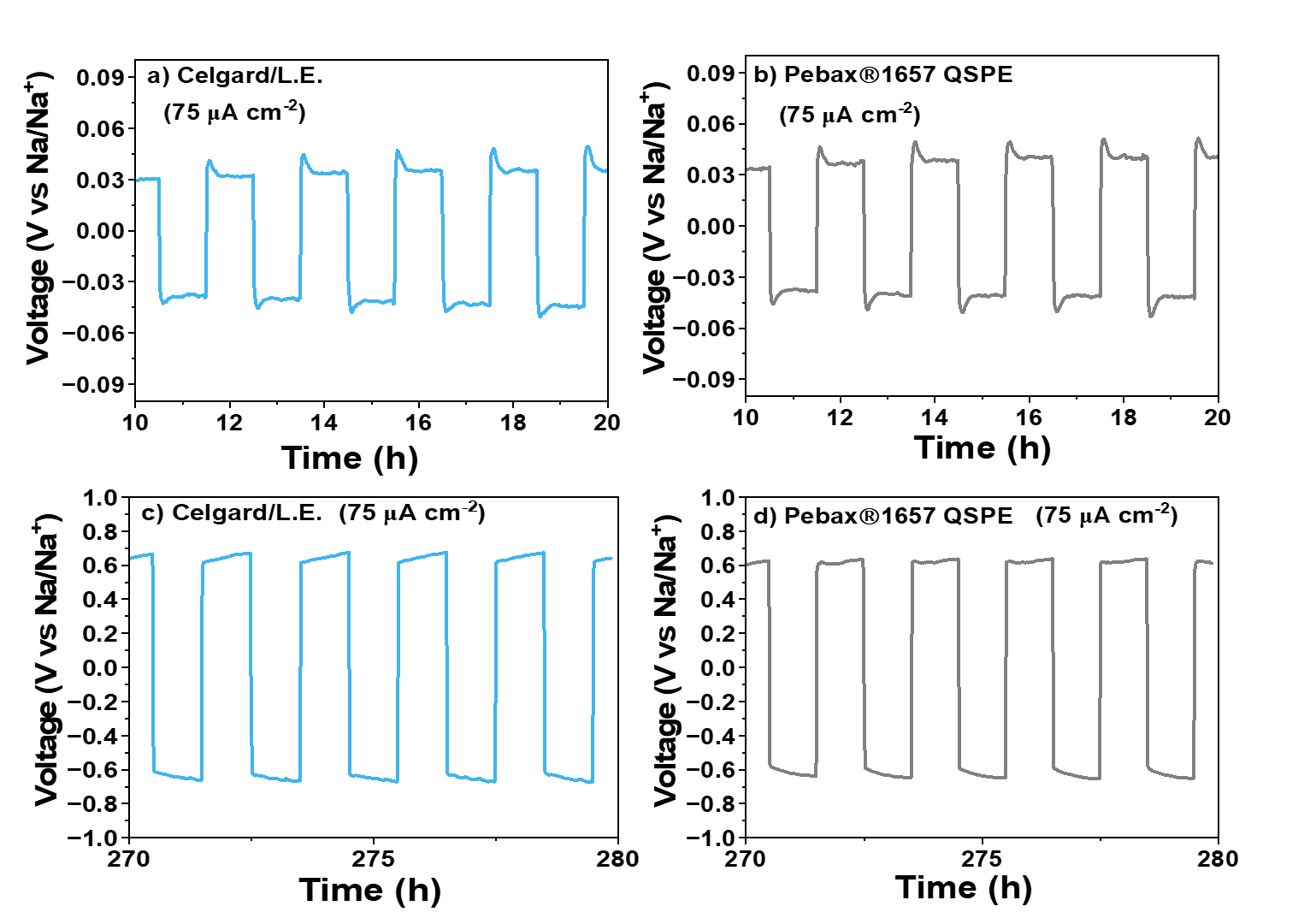


**Figure S11.** Voltage profiles of Na|Na symmetrical cells using Celgard/L.E (a and c) Pebax®1657 QSPE (b and f) for different stags long-term galvanostatic cycling at current density (75 µA cm^-2^), respectively.


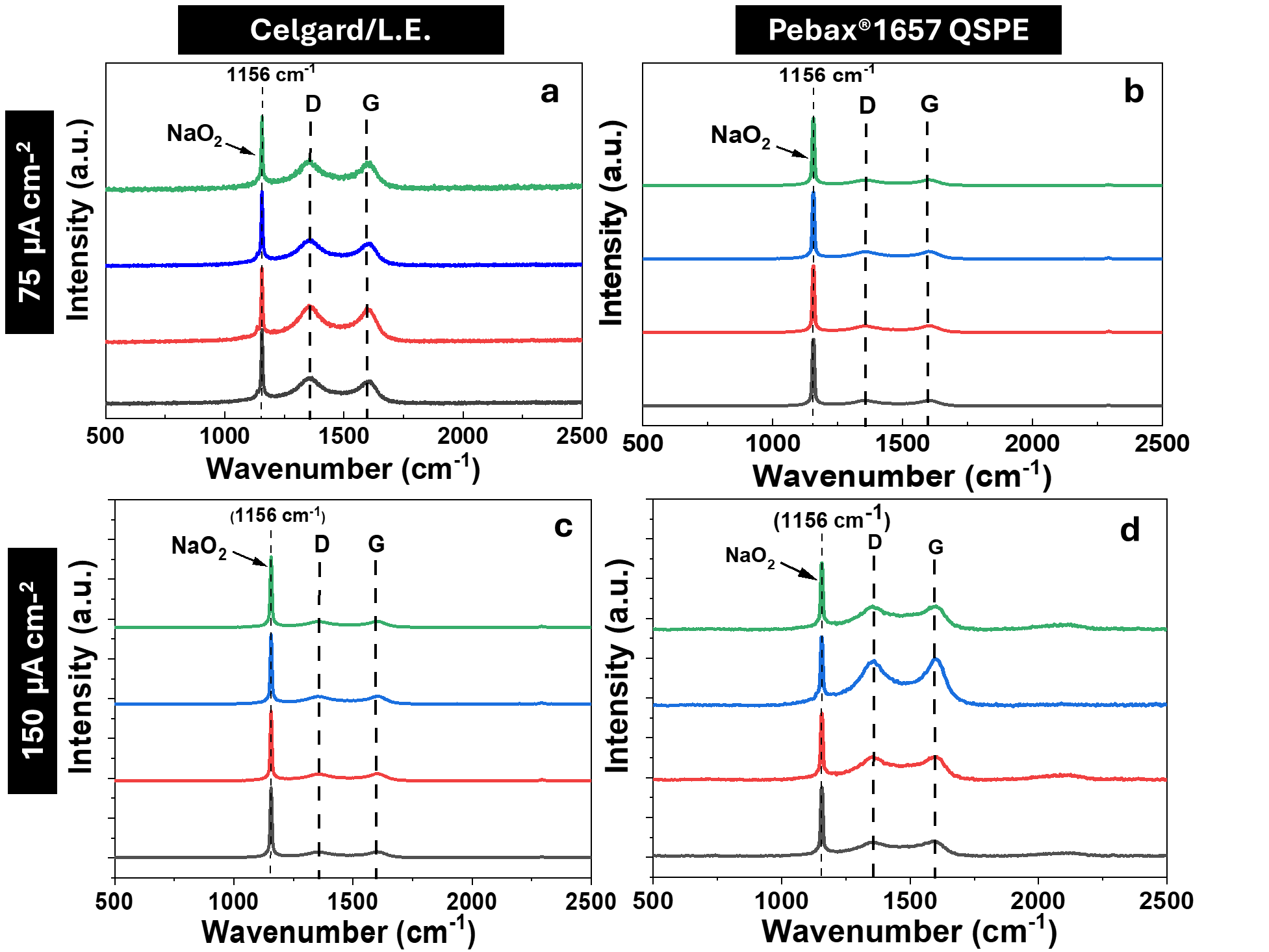


**Figure S12.** Raman spectra of the air cathodes discharged at a-b)75 µA cm^-2^ and c-d) 150 µA cm^-2^, for the Swagelok-type Na-O_2_ batteries using a-c) Celgard/L.E. and b-d) Pebax®1657 QSPE.


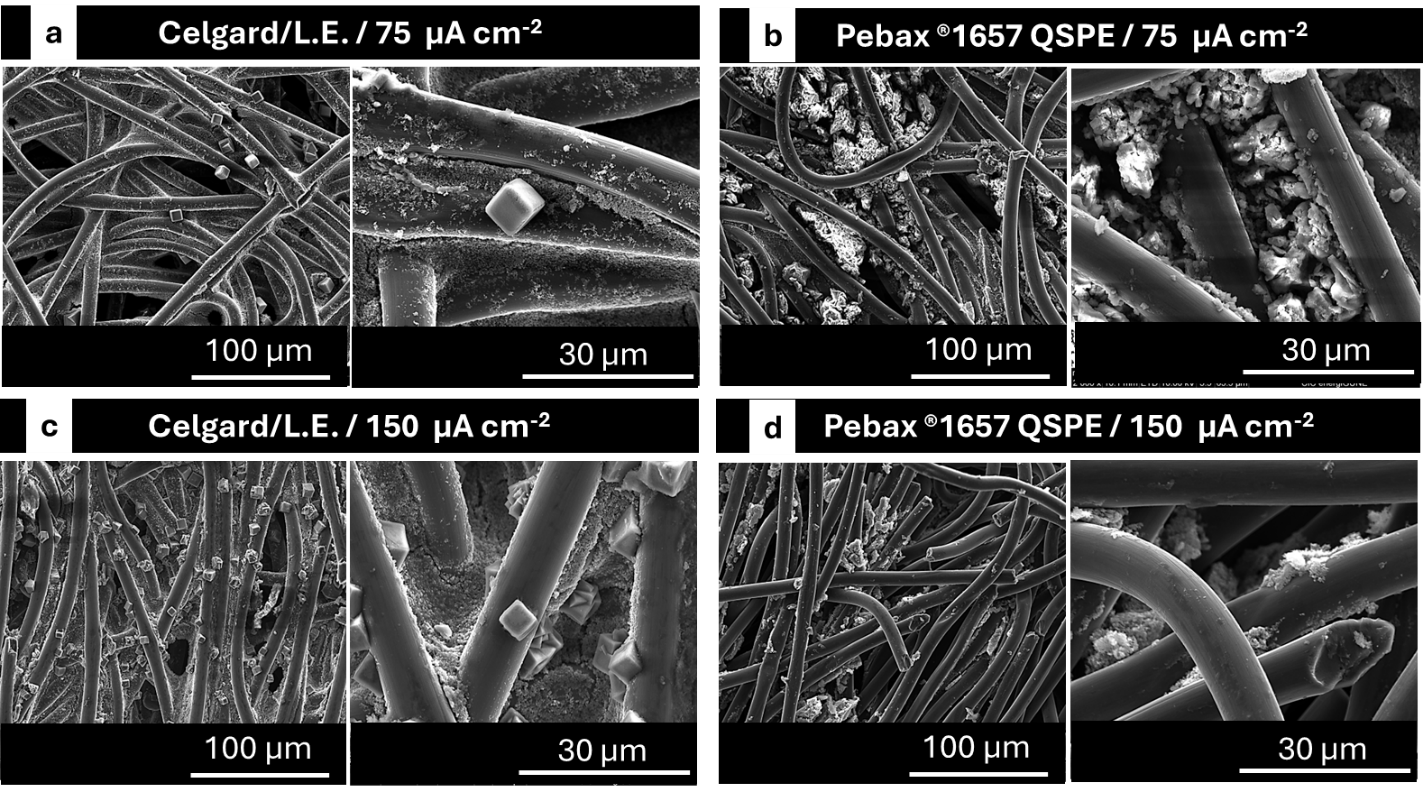


**Figure S13.** SEM imaging of the cathode/electrolyte interface after discharge at different current densities; a-b)75 µA cm^-2^ and c-d) 150 µA cm^-2^, for the batteries assembled using a) Celgard/L.E. and Pebax®1657 QPE systems.


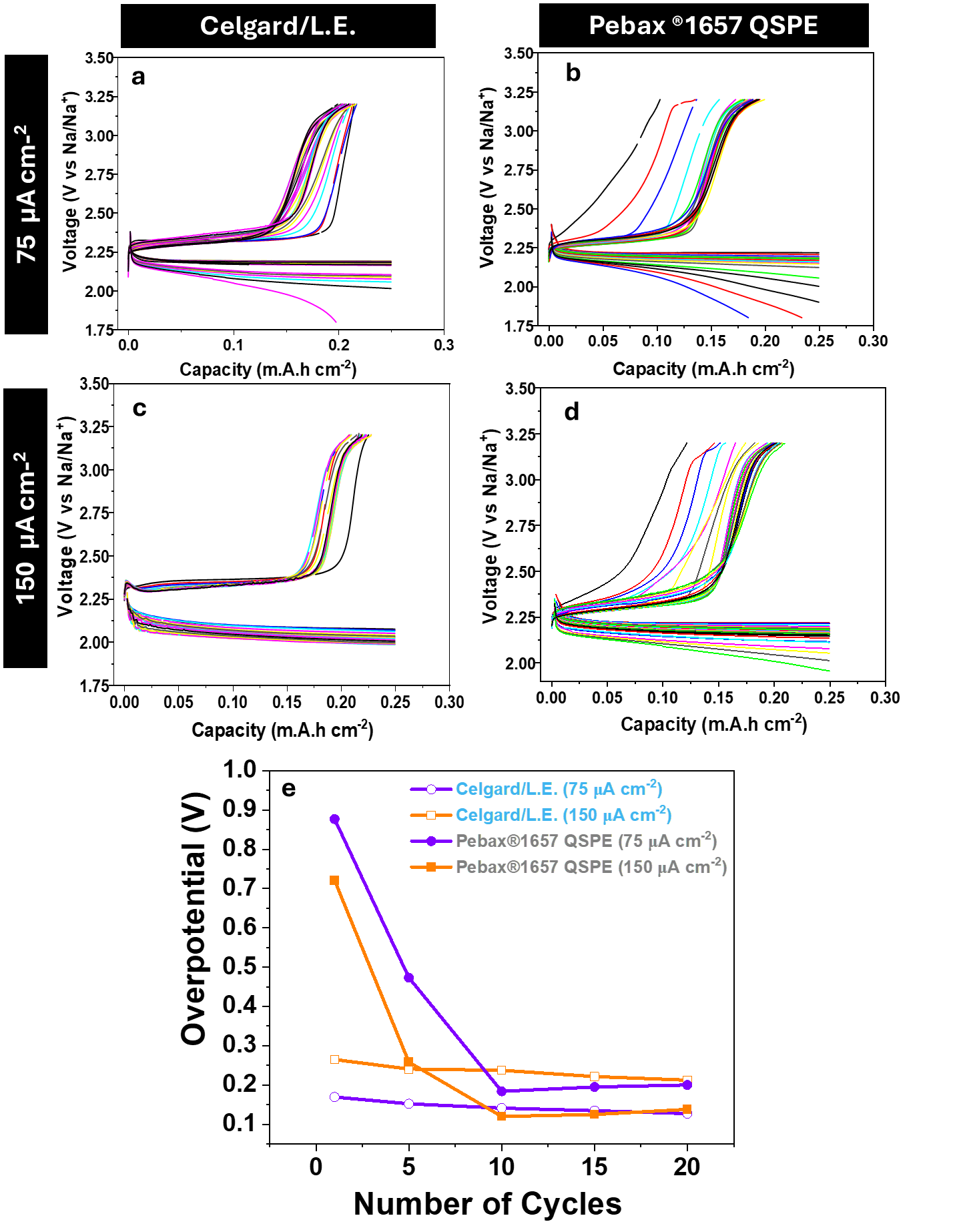


**Figure S14.** Galvanostatic discharge/charge (shallow cyclability) curves at current densities (75 µA cm^−2^ and 150 µA cm^−2^) for the Swagelok-type Na-O_2_ batteries assembled cells using Celgard/L.E. (a and c) and Pebax®1657 QSPE (b and d) to a limited capacity (0.25 mAh cm^-2^) and Cut-off potential (1.8 V). The charge overpotential evolution during cycling for both Celgard/L.E. and Pebax®1657 QSPE at the two current densities (75 μA cm^-2^ and 150 μA cm^-2^) (e). Both polymers were immersed in 1 M NaTFSI/diglyme inside the glovebox.


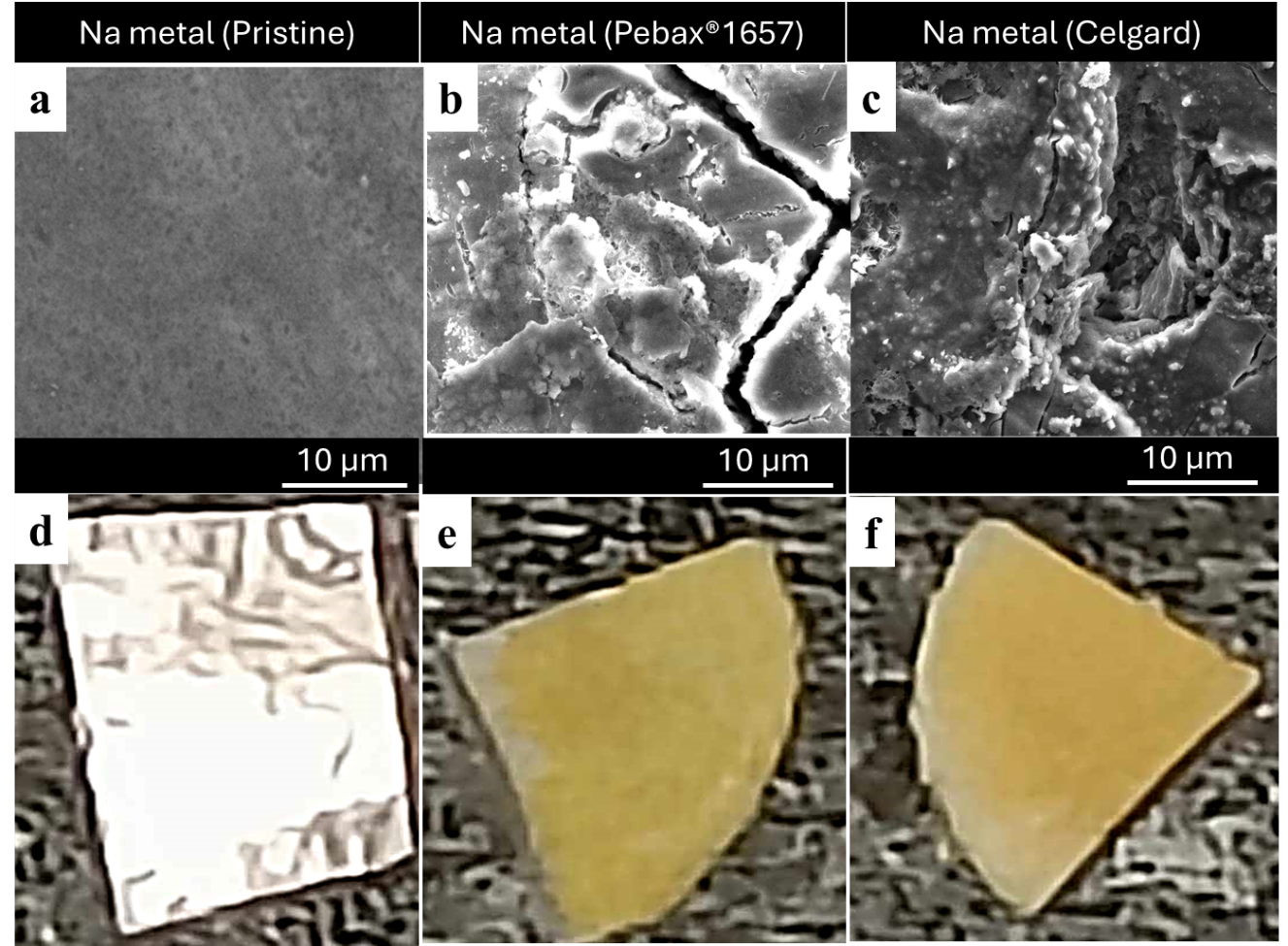


**Figure S15.** SEM imaging and digital photos of the pristine Na anode (a, d) and Na anode for the discharged Swagelok-type Na-O_2_ batteries assembled cells at current density (75 µA cm^-2^) using Pebax®1657 QSPE (b, e) and Celgard/L.E. (c, f).

**Table S1.** The assigned chemical-shifts for the liquid electrolyte (NaTFSI/diglyme) as observed in the NMR spectra.

| Resonance Signal (ppm) |  | ^1^H-NMR | | | Assignment |
| --- | --- | --- | --- | --- | --- |
| δ = 2.55 - 2.65 ppm |  | | (3H)  (singlet, s) | | methyl group protons (-CH_3_) in diglyme |
| δ = 2.75-2.85 ppm |  | | (2H)  (multiplet, m) | | methylene groups (-OCH_2_) in diglyme |
| δ = 2.5 ppm |  | | (6H)  (singlet, s) | | DMSO-d6 |
| Resonance Signal (ppm) |  | | | **^23^Na-NMR** | **Assignment** |
| δ = -5 ppm |  | | | Na⁺ ions  (singlet, s) | NaTFSI salt |
| Resonance Signal (ppm) |  | | | **^19^F-NMR** | **Assignment** |
| δ = -79.8 ppm |  | | | TFSI⁻ anion  (singlet, s) | NaTFSI salt |

**Table S2.** The assigned FTIR bands of Pebax®1657 QSPE, Pebax®1657 membrane, NaTFSI/diglyme electrolyte, Celgard membrane, and Celgard/L.E, respectively.

| \| \| Pebax®1657 QSPE \| \| \| \| \| --- \| --- \| --- \| --- \| \| Vibrational modes \|  \| **Wavenumbers (cm^-1^)** \| \| \| N-H stretching \|  \| \| 3300 \| \| N-H bending \|  \| \| 1541 \| \| C=O stretching \|  \| \| 1640 \| \| C-N stretching \|  \| \| 1733 \| \| C-O stretching \|  \| \| 1104 (come from Pebax®1657 membrane) \| \| C-O stretching \| \|  \| \| 1070 (come from NaTFSI/diglyme electrolyte) \| \| \| \| \| --- \| --- \| --- \| --- \| --- \| --- \| --- \| --- \| --- \| --- \| --- \| --- \| --- \| --- \| --- \| --- \| --- \| --- \| --- \| --- \| --- \| --- \| --- \| --- \| --- \| --- \| --- \| --- \| --- \| --- \| --- \| --- \| --- \| --- \| --- \| --- \| \| -CH_2_ stretching \|  \| 2900 \| \| -CH_2_ bending \|  \| 1350 \| \| C-O stretching \|  \| 1082 \| \| S=O (sulfonyl group) asymmetric stretching \|  \| 1456 \| \| S=O (sulfonyl group) symmetric stretching \|  \| 1186 \| \| CF₃ stretching \|  \| 1103 \| \| S-N stretching \|  \| 859 \| | | | |
| --- | --- | --- | --- | --- | --- | --- | --- | --- | --- | --- | --- | --- | --- | --- | --- | --- | --- | --- | --- | --- | --- | --- | --- | --- | --- | --- | --- | --- | --- | --- | --- | --- | --- | --- | --- | --- | --- | --- | --- | --- | --- | --- | --- | --- | --- | --- | --- | --- | --- | --- | --- | --- | --- | --- | --- | --- | --- | --- | --- | --- |
| Pebax®1657 membrane | | | |
| Vibrational modes |  | **Wavenumbers (cm^-1^)** | |
| N-H stretching |  | | 3294 |
| N-H bending |  | | 1535 |
| CH_2_ stretching |  | | 2900 |
| C=O stretching |  | | 1635 |
| C-N stretching |  | | 1725 |
| C-O stretching |  | | 1098 |
| NaTFSI/diglyme electrolyte | | | |
| Vibrational modes |  | | **Wavenumbers (cm^-1^)** |
| -CH_2_ stretching |  | | 2885 |
| -CH_2_ bending |  | | 1350 |
| C-O stretching |  | | 1070 |
| S=O (sulfonyl group) asymmetric stretching |  | | 1456 |
| S=O (sulfonyl group) symmetric stretching |  | | 1186 |
| CF₃ stretching |  | | 1103 |
| S-N stretching |  | | 859 |
| Celgard membrane | | | |
| Vibrational modes |  | | **Wavenumbers (cm^-1^)** |
| -CH_2_ stretching |  | | 2900 |
| CH₃ symmetric deformation |  | | 1460 |
| -CH₂ bending |  | | 1376 |
| C-C stretching |  | | 971 |
| Celgard/L.E. | | | |
| Vibrational modes |  | | **Wavenumbers (cm^-1^)** |
| -CH_2_ stretching |  | | 2900 |
| CH₃ symmetric deformation |  | | 1460 |
| -CH₂ bending |  | | 1376 |
| C-C stretching |  | | 971 |
| -CH_2_ bending |  | | 1350 |
| C-O stretching |  | | 1070 |
| S=O (sulfonyl group) asymmetric stretching |  | | 1456 |
| S=O (sulfonyl group) symmetric stretching |  | | 1186 |
| CF₃ stretching |  | | 1103 |
| S-N stretching |  | | 859 |

**Table S3.** The assigned Raman bands of the NaTFSI/diglyme electrolyte, Pebax®1657 membrane, Pebax®1657 QSPE, and Pebax®1657 QSPE, respectively.

| **NaTFSI/diglyme electrolyte** | |
| --- | --- |
| **Vibrational modes** | **Raman peaks (cm^-1^)** |
| TFSI⁻ (S-N-S bending) | ~743 |
| Diglyme (C-O-C stretch) | ~845 |
| TFSI⁻ (CF_3_ stretch) | ~ 1240 |
| TFSI⁻ (SO_2_ stretch) | ~ 1260 |
| Diglyme (CH_2_ stretch) | ~1450 |
| **Pebax®1657** | |
| **Vibrational modes** | **Raman peaks (cm^-1^)** |
| PA (C-N bending) | ~930 |
| PEO (C-O stretching) | ~ 830, 1060, 1126 and 1280 |
| PA (C-N stretching) | ~1445 |
| PEO (CH_2_ stretching) | ~1465 |
| PA (C=O stretching) | ~1640 |
| **Pebax QSPE®1657** | |
| **Vibrational modes** | **Raman peaks (cm^-1^)** |
| TFSI⁻ (S-N-S bending) | broadened ~743 |
| Diglyme (C-O-C stretch) | broadened ~845 |
| TFSI⁻ (CF_3_ stretch) | broadened ~1240 |
| TFSI⁻ (SO_2_ stretch) | broadened ~1260 |
| Diglyme (CH_2_ stretch) | broadened ~1450 |
| PA (C-N bending) | broadened ~930 |
| PEO (C-O stretching) | broadened/Shifted  ~ 850, 1065, 1135 and 1287 |
| PA (C-N stretching) | Shifted/broadened  ~1450 |
| PEO (CH_2_ stretching) | broadened ~1465 |
| PA (C=O stretching) | broadened /dismissed ~1640 |
| **Pebax QSPE®1657 (Cycling)** | |
| **Vibrational modes** | **Raman peaks (cm^-1^)** |
| TFSI⁻ (S-N-S bending) | Shifted/broadened ~737 |
| Diglyme (C-O-C stretch) | Highly broadened /dismissed ~845 |
| TFSI⁻ (CF_3_ stretch) | Highly broadened /dismissed ~1240 |
| TFSI⁻ (SO_2_ stretch) | Highly broadened /dismissed ~1260 |
| Diglyme (CH_2_ stretch) | Highly broadened /dismissed ~1450 |
| PA (C-N bending) | Highly broadened /dismissed ~930 |
| PEO (C-O stretching) | Highly broadened /dismissed ~ 850, 1065, 1135 and 1287 |
| PA (C-N stretching) | Highly broadened /dismissed ~1450 |
| PEO (CH_2_ stretching) | Highly broadened /dismissed ~1465 |
| PA (C=O stretching) | Highly broadened /dismissed ~1640 |

**Table S4.** Comparison of the electrochemical performance of this work with reported polymer electrolytes for Na-O_2_ batteries in the literature.

| **Electrolyte** | **Air Cathode**  **[loading (mg) and area (cm^2^)]** | **Full capacity** | **Cycling performance** | **Voltage window**  **(V)** | **Cycling overpotential**  **(V)** | **Coulombic efficiency (C.E.)** | **Gravimetric**  **Energy Density** | **Ref.** |
| --- | --- | --- | --- | --- | --- | --- | --- | --- |
| Ionogel electrolyte (16.6 mol% NaTFSI in (C_4_mpyr][TFSI]/ PEGDA). | CNFs mat  [1.58 mg cm^-2^] | 0.17 mAh cm^-2^ at 25 µA cm^-2^  Cut off: 1.6V | 2 cycles (at 25 µA cm^−2^ and 0.05 mAh cm^−2^) + 2 cycles (at 25 µA cm^−2^ and 25 µAh cm^−2^) | 1.6-3.2 | 0.8-1 | ~100% | 213.04 Wh/kg | Ha et al., ^[1]^ |
| Quasi-solid-state electrolyte (PVDF-HFP/ 4% SiO_2_−NaClO_4_-TEGDME) | Super P-supported in carbon paper  ** | * | 80 cycles (at 200 mA g^-1^ and 1000 mAh g^-1^) | 1.8-3 | 0.5  Constant over cycling | ~97% | * | Wang et al., ^[2]^ |
| PEO/NaTFSI/ 25 wt.% NZSP/ (1M NaClO_4_ in TEGDME) | CNT  [0.5 mg cm^-2^] | 4789 mAh g^−1^ at 100 mA g^-1^ or 2.39 mAh cm^-2^ at 44 µA cm^-2^  Cut off: 1.5V | 25 cycles (at 100 mA g^−1^ and 500 mAh g^−1^ or 44 µA cm^-2^ and 0.22 mAh cm^-2^) | 1.6-4.5 | 0.3-1.3 | ~100% | 340.76 Wh/kg | Iputera et al., ^[3]^ |
| PVB-PDADMATFSI-PVB block copolymer/ (NaTFSI/DGME/C_4_mpyrTFSI). | CNFs mat  [Estimated mass 1.58 mg cm^-2^] | 1.59 mAh cm⁻² at 75 µA cm⁻²  Cut off: 1.6V | * | 1.6-3.2 | * | * | 182.21 Wh/kg | Stigliano et al., ^[4]^ |
| Pebax®1657 QSPE (1M NaTFSI/diglyme) | H23C6 carbon paper  [15: 1.13] | 2.60 mAh cm⁻² at 75 µA cm⁻² & 2.11 mAh cm⁻² 150 µA cm⁻²  Cut off:1.8V | 25 cycles at (75 µA cm^-2^ and 0.25 mAh cm^−2^) or 35 cycles at (150 µA cm^-2^ and capacity 0.25 mAh cm^−2^) | 1.8-3.2 | < 0.2 after 8 cycles stabilization | ~85% | 377.42 Wh/kg | **This work** |

*Not available

** The weight of the support was not considered for calculations so gravimetric data is probably overestimated.

**The calculation for the gravimetric energy density:**

The theoretical gravimetric energy density of Na-O_2_ batteries depends on the chemical composition of the discharge product. For instance, in the case of sodium peroxide (Na_2_O_2_) as the discharge product, the theoretical energy density is 1605 Wh/kg, whereas in the case of sodium superoxide (NaO_2_) a slightly lower value (1105 Wh/kg) is expected.

The gravimetric energy density is calculated using the formula ^[5]^:

Gravimetric Energy Density (Wh/kg) = Theoretical Capacity (mAh/g) x Average Voltage (V)/ Total Mass of Active Materials (g).

Gravimetric Energy Density (Wh/kg) = Areal Capacity (mAh/cm^2^) x Average Voltage (V)/ Total Mass of Active Materials (g/cm^2^).

**Where:** Areal Capacity: capacity obtained from the experimental data, Average Voltage: The average discharge voltage (plateau voltage) from experimental data, Mass of Active Materials: Considering both active material and carbon substrate mass in cathode.

**1. Ionogel electrolyte (CNFs mat)**

Areal Capacity: 0.17 mAh/cm² and voltage: 1.98 V

Total Mass: 1.58 mg (CNFs) free standing without subtract = 1.58 mg/cm² = 0.00158 g/cm²

Energy Density: 0.17×1.98/ 0.00158 = 213.04 Wh/kg

**2. PEO/NaTFSI/NZSP / CNT**

Areal Capacity: 2.39 mAh/cm² and Voltage: 2.21 V

Total Mass: 0.5 mg (CNT) + 15 mg (substrate) = 15.5 mg/cm² = 0.0155 g/cm²

Energy Density: 2.39×2.21/ 0.0155 ​= 340.76 Wh/kg

**3. PVB-PDADMATFSI-PVB / CNFs Mat**

Areal Capacity: 1.59 mAh/cm² and Voltage: 1.90 V

Total Mass: 1.58 mg (CNFs) + 15 mg (substrate) = 16.58 mg/cm² = 0.01658 g/cm²

Energy Density:1.59×1.90/0.01658 = 182.21 Wh/kg

**4. Pebax®1657 QSPE**

Areal Capacity: 2.60 mAh/cm² and volage= 2.25 V

Total Mass: 15 mg (substrate only, assuming negligible electrolyte mass) = 0.015 g/cm²

Energy Density: 2.60 × 2.25/0.0155 = 377.42 Wh/kg

**Table S5.** Comparison of the electrochemical performance of this work with reported polymer electrolytes for Na ion batteries in the literature.

| **Electrolyte System** | **t_Na+_** | **Ionic Conductivity (S cm^-1^)** | **Temperature** | **Oxidation Onset Potential**  **(V vs. Na/Na^+^) (V)** | **Ref.** |
| --- | --- | --- | --- | --- | --- |
| PEO/PNaMTFSI | > 0.83 | 7.74 x 10^-5^ | 85 °C | 3.5-4.5 | Martínez et al.,^[6]^ |
| PSTB-based GPE (PSP-GPE) | 0.88 | 1 x 10^-4^ | RT. | 4.5 | Wang et al.,^[7]^ |
| PVDF-HFP-based GPE (PSIL70) | 0.27 | 1.9 x 10^-3^ | RT. | 4.2 | Mishra et al,.^[8]^ |
| NASICON-based fillers | 0.57 | 2.78 x 10^-3^ | RT. | * | Mei et al., ^[9]^ |
| PEO/Na_3_SbS_4_ | 0.49 | 1.33 x 10^-4^ | RT. | * | Lu et al., ^[10]^ |
| NaPTAB-SGPE | 0.91 | 9.4 x 10^-5^ | RT. | 5.2 | Yang et al.,^[11]^ |
| PVDF-HFP/PMMA-based GPE | 0.2 | 5.8 x 10^-3^ | RT. | 5 | Patel et al., ^[12]^ |
| Pebax QSPE | 0.4 | 6.57 x 10^-4^ | RT. | 4.69 | **This work** |

*Not available

**References**

[1] T. A. Ha, A. Fdz De Anastro, N. Ortiz-Vitoriano, J. Fang, D. R. MacFarlane, M. Forsyth, D. Mecerreyes, P. C. Howlett, C. Pozo-Gonzalo, *The Journal of Physical Chemistry Letters* **2019**, *10*, 7050-7055. <https://doi.org/10.1021/acs.jpclett.9b02947>.

[2] J. Wang, Y. Ni, J. Liu, Y. Lu, K. Zhang, Z. Niu, J. Chen, *ACS Central Science* **2020**, *6*, 1955-1963. <https://doi.org/10.1021/acscentsci.0c00849>.

[3] K. Iputera, C.-F. Tsai, J.-Y. Huang, D.-H. Wei, R.-S. Liu, *ACS Applied Materials & Interfaces* **2024**. <https://doi.org/10.1021/acsami.4c04613>.

[4] P. L. Stigliano, A. Gallastegui, C. Villacis-Segovia, M. Amores, A. Kumar, L. A. O’Dell, J. Fang, D. Mecerreyes, C. Pozo-Gonzalo, M. Forsyth, in *Batteries, Vol. 10*, 2024.

[5] K. Song, D. A. Agyeman, M. Park, J. Yang, Y.-M. Kang, *Advanced Materials* **2017**, *29*, 1606572. <https://doi.org/https://doi.org/10.1002/adma.201606572>.

[6] J. L. Olmedo-Martínez, A. Fdz De Anastro, M. Martínez-Ibañez, A. J. Müller, D. Mecerreyes, *Energy & Fuels* **2023**, *37*, 5519-5529. <https://doi.org/10.1021/acs.energyfuels.2c04296>.

[7] P. Wang, H. Zhang, J. Chai, T. Liu, R. Hu, Z. Zhang, G. Li, G. Cui, *Solid State Ionics* **2019**, *337*, 140-146. <https://doi.org/https://doi.org/10.1016/j.ssi.2019.04.022>.

[8] R. Mishra, S. K. Singh, H. Gupta, R. K. Tiwari, D. Meghnani, A. Patel, A. Tiwari, V. K. Tiwari, R. K. Singh, *Energy & Fuels* **2021**, *35*, 15153-15165. <https://doi.org/10.1021/acs.energyfuels.1c02114>.

[9] W. Mei, X. Wang, Y. Wang, J. Chen, Z. Mao, D. Wang, *Journal of Solid State Chemistry* **2021**, *302*, 122459. <https://doi.org/https://doi.org/10.1016/j.jssc.2021.122459>.

[10] Y. Lu, L. Li, Q. Zhang, Y. Cai, Y. Ni, J. Chen, *Chemical Science* **2022**, *13*, 3416-3423. <https://doi.org/10.1039/D1SC06745A>.

[11] L. Yang, Y. Jiang, X. Liang, Y. Lei, T. Yuan, H. Lu, Z. Liu, Y. Cao, J. Feng, *ACS Applied Energy Materials* **2020**, *3*, 10053-10060. <https://doi.org/10.1021/acsaem.0c01756>.

[12] M. Patel, K. Mishra, N. A. Chaudhary, V. Madhani, J. J. Chaudhari, D. Kumar, *RSC Advances* **2024**, *14*, 14358-14373. <https://doi.org/10.1039/D4RA01615G>.
